# An Exoneuromusculoskeleton for Self-Help Upper Limb Rehabilitation After Stroke

**DOI:** 10.1089/soro.2020.0090

**Published:** 2022-02-14

**Authors:** Chingyi Nam, Wei Rong, Waiming Li, Chingyee Cheung, Wingkit Ngai, Tszching Cheung, Mankit Pang, Li Li, Junyan Hu, Honwah Wai, Xiaoling Hu

**Affiliations:** ^1^ Department of Biomedical Engineering, The Hong Kong Polytechnic University, Hong Kong, China.; ^2^ Industrial Centre, The Hong Kong Polytechnic University, Hong Kong, China.; ^3^ Institute of Textiles and Clothing, The Hong Kong Polytechnic University, Hong Kong, China.

**Keywords:** stroke rehabilitation, robot, pneumatic muscle, neuromuscular electrical stimulation, exoskeleton

## Abstract

This article presents a novel electromyography (EMG)-driven exoneuromusculoskeleton that integrates the neuromuscular electrical stimulation (NMES), soft pneumatic muscle, and exoskeleton techniques, for self-help upper limb training after stroke. The developed system can assist the elbow, wrist, and fingers to perform sequential arm reaching and withdrawing tasks under voluntary effort control through EMG, with a lightweight, compact, and low-power requirement design. The pressure/torque transmission properties of the designed musculoskeletons were quantified, and the assistive capability of the developed system was evaluated on patients with chronic stroke (*n* = 10). The designed musculoskeletons exerted sufficient mechanical torque to support joint extension for stroke survivors. Compared with the limb performance when no assistance was provided, the limb performance (measured as the range of motion in joint extension) significantly improved when mechanical torque and NMES were provided (*p* < 0.05). A pilot trial was conducted on patients with chronic stroke (*n* = 15) to investigate the feasibility of using the developed system in self-help training and the rehabilitation effects of the system. All the participants completed the self-help device-assisted training with minimal professional assistance. After a 20-session training, significant improvements were noted in the voluntary motor function and release of muscle spasticity at the elbow, wrist, and fingers, as indicated by the clinical scores (*p* < 0.05). The EMG parameters (*p* < 0.05) indicated that the muscular coordination of the entire upper limb improved significantly after training. The results suggested that the developed system can effectively support self-help upper limb rehabilitation after stroke. ClinicalTrials.gov Register Number NCT03752775.

## Introduction

Upper limb motor deficits are noted in >80% of stroke survivors,^1,2^ who require continuous long-term physical rehabilitation to reduce upper limb impairments.^3,4^ Restoration of poststroke limb function requires intensive repeated training of the paralyzed limb^5,6^ with maximized voluntary motor effort^7,8^ and minimized compensatory motions in close-to-normal muscular coordination.^8,9^ However, long-term poststroke rehabilitation is challenging because of the expanding stroke population and insufficiency of professional staff worldwide.^10,11^ Effective rehabilitation methods with potential for self-help training by stroke survivors are urgently required to improve the independency of stroke survivors and decrease the burden on the health care system. Suitable technologies for these methods are currently lacking.^11,12^

Various rehabilitation robots have been developed to assist the labor-intensive process of physical poststroke training, with main advantages of higher dosage and lower cost compared with traditional “one-to-one” manual physical therapy.^13^ However, these robots are large equipment powered by alternating current (AC) that require professional operation in a clinical environment with limited access to outpatients. Mobile exoskeletons are an emerging technology with wearable application. These exoskeletons are powered by portable batteries and have potential for user-independent self-help rehabilitation that can be accessed anytime, even in unconventional environments (e.g., at home).^12,14,15^ However, currently available upper limb exoskeletons, which are composed of rigid materials and actuated by electrical motors, are constrained by their heavy weight and low torque-to-weight ratio, which limit their user-independent applications. These exoskeletons require high-power consumption because their actuations must generate sufficient torque to support paralyzed limbs as well as the weight of the system worn on the body. Thus, most exoskeletons require AC supply,^11,15,16^ which triggers electrical safety concerns for user-independent usage.

Furthermore, the body/device integration is neither stable nor comfortable in current rigid exoskeletons, with misalignment or migration occurring during repeated practice mainly because of the non-negligible weights mounted onto the paretic limb.^11,14^ Misalignments with additional loads deteriorate abnormal muscular coordination in the paralyzed upper limb, which undermines the rehabilitative potential of the aforementioned systems.^17,18^ Therefore, most rigid exoskeletons for poststroke upper limb rehabilitation are still used under the close assistance of professionals in clinical environments, and their rehabilitation effects in user-independent operations are unclear.

With the introduction of soft materials in mechanical actuation, soft robotic equipment has been designed using easily deformable materials with light and flexible actuators that conform to human body contours^19–22^ so as to achieve superior body/device integration to that provided by rigid robotic equipment. Three main types of actuation systems, namely cable, hydraulic, and pneumatic systems, are used in current wearable soft robots.^21^ Cable systems used cables with desired tension attached to a target limb for flexion/extension.^11,23^ The cable-driven upper limb exoskeletons usually have a lightweight design with low inertia in the wearable part accommodating possible joint misalignment between the paretic limb and the exoskeleton.^23^ However, the cable is driven by electric motors with gears/pulleys, leading to an increment of complexity and overall weight of the whole assembly.^23^ Hydraulic systems are powered by hydraulic pressure, and able to produce greater torque compared with the actuators in cable and pneumatic systems.^11,23,24^ However, few hydraulic systems have been developed for upper limb, because they are relatively heavy and complex in the design, requiring additional space to accommodate the fluid and to prevent leakages under pressure.^11,16,23^

In contrast, pneumatic systems (pneumatic muscles) are the most commonly adopted actuation for the upper limb.^21,23^ Pneumatic exoskeletons have high torque-to-weight ratios because of the low weight of the wearable part actuated by air.^21,25–29^ However, pneumatic systems are usually bulky and slow in power transmission from pressure to torque during air inflation by compressors for needed air volume and pressure compared with electrical motor actuation in rigid exoskeleton to achieve equivalent mechanical outputs (e.g., joint torque).^23,30^ Large and high-power compressors connected to the pneumatic muscles constrain these devices for user-independent applications.^21^ Thus, a novel lightweight mechanical design is required to achieve optimized body/device integration with fast power transmission, high torque-to-weight ratios, and low-power consumption for user-independent self-help rehabilitation.

Neuromuscular electrical stimulation (NMES), proposed for upper limb rehabilitation,^31,32^ can activate the contraction of impaired muscles to generate limb movement^31,32^ and effectively enhance the muscle force and sensory feedback for motor relearning after stroke.^33^ However, controlling motion kinematics, such as the range of motion (ROM) and trajectory, by using NMES alone is difficult because of the limited stimulating precision in fine motor control.^34^ Recently, NMES has been combined with mechanical robots in poststroke training.^35^ The combined NMES-robot treatment is more effective than treatment involving the use of only NMES or only a robot in upper limb rehabilitation, particularly in improving muscular coordination by reducing muscular compensation.^36^ The integration of NMES into a robot can trigger the biological actuation of target muscles to reduce the demand of mechanical support from the robot part.^11^ However, little has been done on the integration of NMES with mobile exoskeletons or soft robots.

In this study, we designed a multi-integrated robotic system that combines the NMES, soft pneumatic muscle, and exoskeleton techniques, namely exoneuromusculoskeleton, for upper limb rehabilitation after stroke. Mechanical integration between rigid exoskeleton and pneumatic muscle (i.e., exomusculoskeleton) can enable high torque-to-weight ratios with a compact size and fast power transmission. By combining NMES with the exomusculoskeleton (i.e., exoneuromusculoskeleton), the mechanical scale and power requirement of the entire system can be reduced due to the evoked muscular effort. In addition, NMES and mechanical assistance enable the achievement of close-to-normal muscular coordination with minimized compensatory motions. To optimize therapeutic outcomes, electromyography (EMG) of the paralyzed limb has been used to indicate voluntary intentions^37^ to maximize voluntary motor effort during practice for better improvements in voluntary motor functions with longer sustainability compared with those with passive limb motions.^38^

In this study, we designed an EMG-driven exoneuromusculoskeleton to assist the upper limb physical practice at the elbow, wrist, and fingers. The assistive capability of the designed system was evaluated on patients with chronic stroke. The designed system's feasibility of self-help operation and rehabilitation effects were also investigated through a pilot single-group trial.

## Methods

**FIG. 1.**
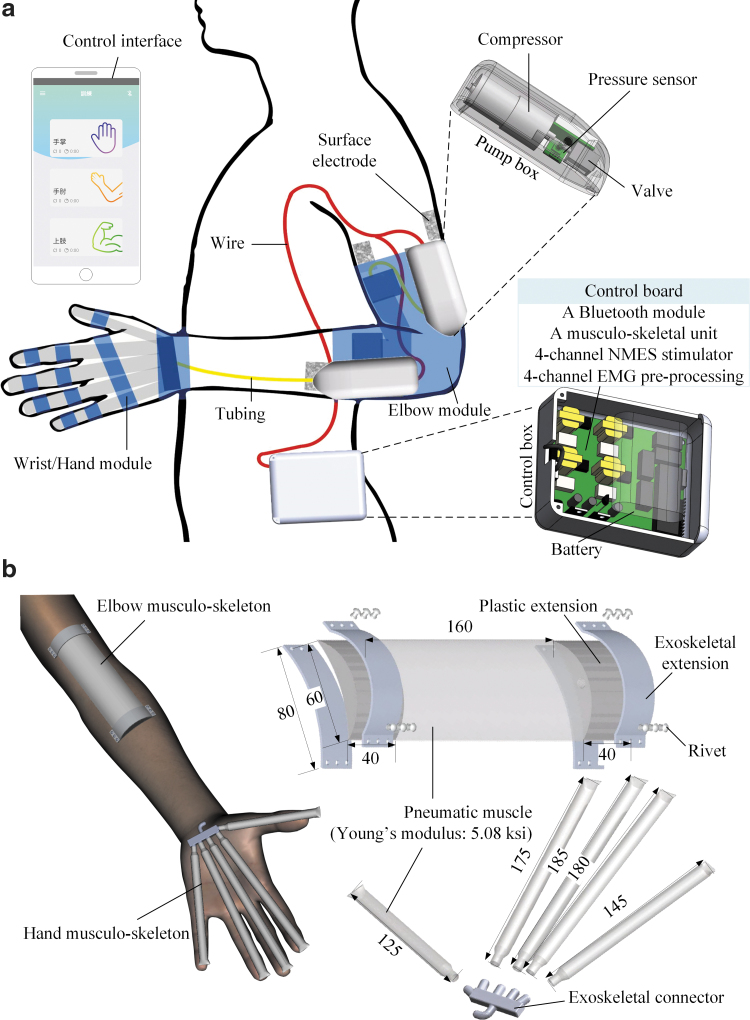
**(a)** Overview of the exoneuromusculoskeleton, with the inner structures of a pump box and the control box. **(b)** Attachment of the musculoskeletons, and structures with dimensions of the elbow musculoskeleton and the hand musculoskeleton (all the dimensions are in millimeters). EMG, electromyography; NMES, neuromuscular electrical stimulation.

The designed exoneuromusculoskeleton (Fig. 1) could be worn on the paretic upper limb of a stroke survivor. The designed system comprised two wearable parts: the elbow (158 g) and wrist/hand (50 g). Both parts were connected to a pump box (80 g) mounted on the upper limb. Moreover, a control box (358 g) that included system control circuits and a rechargeable 12-V battery could be carried on the waist. The developed system can assist a stroke survivor to perform sequential arm reaching and withdrawing tasks, namely (1) elbow extension, (2) wrist extension with the hand open, (3) wrist flexion with the hand closed, and (4) elbow flexion. Real-time control and wireless communication between the control box and a mobile application (app) were achieved on a smartphone through a microprocessor and Bluetooth module.

### System control platform

**FIG. 2.**
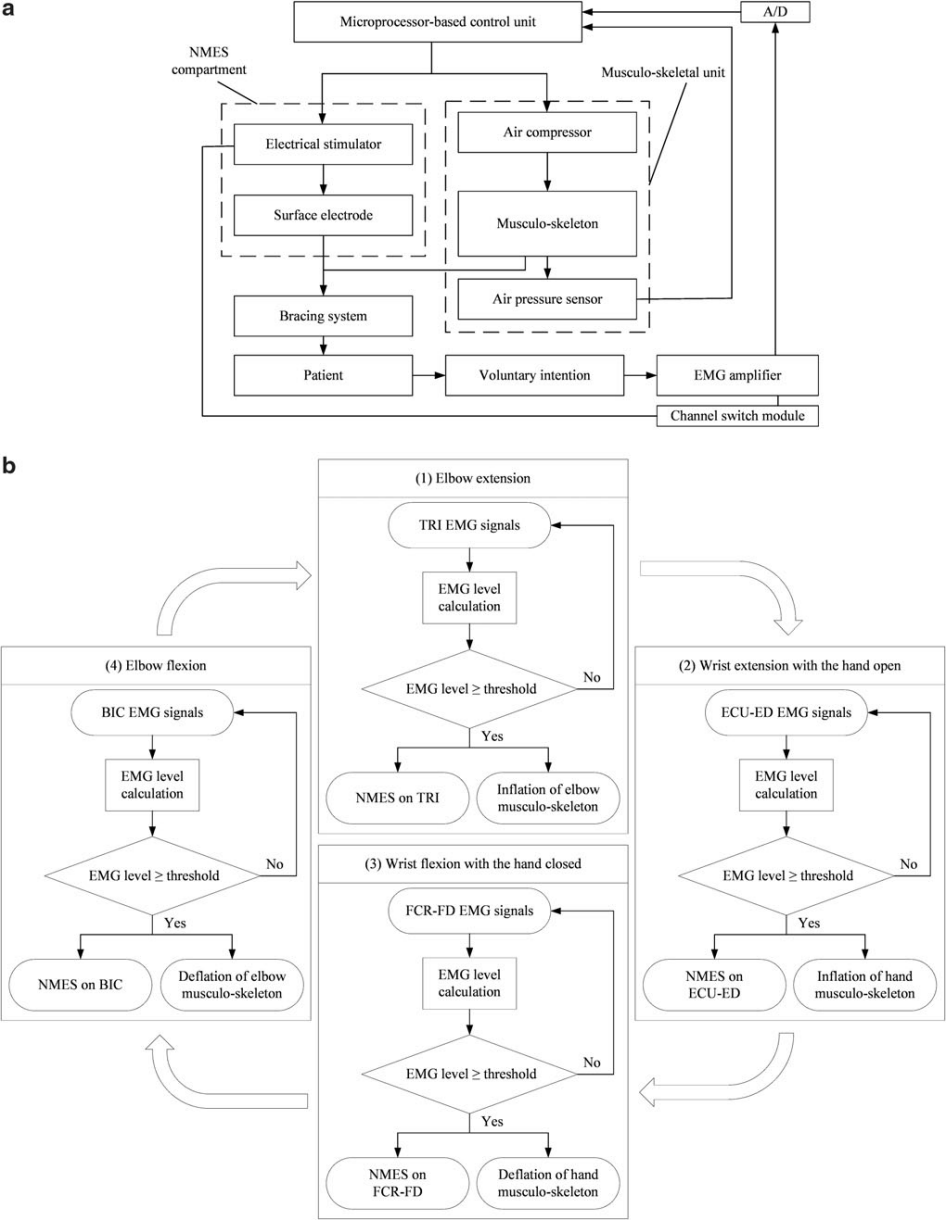
**(a)** The schematic diagram of the control in the EMG-driven exoneuromusculoskeleton, and **(b)** the controlling workflow of the assistance in phasic and sequential limb tasks. BIC, biceps brachii; ECU, extensor carpi ulnaris; ED, extensor digitorum; FCR, flexor carpi radialis; FD, flexor digitorum; TRI, triceps brachii.

Figure 2a depicts the system control diagram of the exoneuromusculoskeletal system. The system comprised a microcontroller unit (MCU), a musculoskeletal unit, an NMES compartment, a channel switch module, and an EMG preprocessing module. The MCU (PIC18F46K22; Microchip Technology, Inc., Chandler, AZ) coordinated with a musculoskeletal unit, 4-channel NMES stimulator, 4-channel EMG preprocessing, and wireless communication with the developed app through a Bluetooth module (Bluetooth HC-05; JMoon Technologies, New Delhi, India).

The musculoskeletal unit comprised elbow module and hand module for providing mechanical assistance. Each module included a related musculoskeleton, connected to a respective miniature air compressor (P54A02R; Oken Seiko Co., Ltd., Tokyo, Japan) with a valve and pressure sensor (BMP series; Adafruit, Inc., New York City, NY). The air compressor was used to inflate the musculoskeleton, which would deflate when the valve was opened. The inflated musculoskeleton provided mechanical torque to a joint during extension and it deflated passively during flexion.

The NMES compartment provided electrical stimulation (square pulse with adjustable pulse width of 0–300 μs, 70 V, 40 Hz)^39^ to the muscle of the biceps brachii (BIC) during elbow flexion, muscle of the triceps brachii (lateral head, TRI) during elbow extension, muscle union of the flexor carpi radialis (FCR) and the flexor digitorum (FD) during wrist flexion with the hand closed, and muscle union of the extensor carpi ulnaris (ECU) and the extensor digitorum (ED) during wrist extension with the hand open.^40^ The activation of the musculoskeletons and NMES were controlled by the EMG signals detected for the BIC muscle, TRI muscle, FCR-FD muscle union, and ECU-ED muscle union in different motion phases. In this study, EMG detection and NMES delivery to a target muscle were performed using a pair of surface electrodes (5 × 5 cm^2^, PALS Neurostimulation Electrodes; Axelgaard Manufacturing Co., Ltd., Fallbrook, CA) connected to an EMG-NMES channel. An electrode pair was placed on the motor point at the muscle belly for achieving effective EMG capture and NMES delivery, as achieved in Muraoka's work.^41^ Because of the close anatomical proximity between the FCR and FD muscles and between the ECU and ED muscles, electrode pairs were located in the common area of the motor point of the two muscle bellies of the FCR-FD and ECU-ED muscle unions.^40^ A channel switch circuit was integrated into each EMG-NMES channel and used to alter the functions between the input of the EMG detection and the output of the NMES through the same electrode pair. This circuit also protected the EMG amplification circuit from the high stimulation voltage.^41,42^ A reference electrode (2 × 3 cm^2^, Blue Sensor N; Ambu, Inc., Ballerup, Denmark) was attached to the skin surface of the olecranon for reducing the common mode noise.

The EMG signals captured using the surface electrodes were first amplified 1000 times (preamplifier: INA 333; Texas Instruments, Inc., Dallas, TX) and filtered from 10 to 500 Hz. These amplified and filtered signals were then sampled using an analog-to-digital converter (AD73360; Analog Devices, Inc., Norwood, MA) with a sampling frequency of 1000 Hz for each EMG channel. Digitized EMG data were retrieved using a digital signal controller (dsPIC33F, 16bit; Microchip Technology, Inc.) for further manipulation by the MCU. After digitization, the EMG signals were full-wave rectified and moving-averaged with 100-ms window to obtain the EMG levels.

### EMG-driven control

EMG-triggered control was adopted in this study.^43,44^ In other words, voluntary EMG from a target driving muscle was used to initiate assistance from the developed system. Once the EMG level of a driving muscle or muscle union reached a preset threshold, exoneuromusculoskeletal assistance (i.e., musculoskeleton and NMES) was initiated and continuously provided during an entire motion phase. In each motion phase, a patient was also required to exert the residual voluntary effort, together with the exoneuromusculoskeletal assistance to achieve the desired motion. The controlling workflow of the EMG-driven exoneuromusculoskeleton assisted in phasic and sequential limb tasks is shown in Figure 2b. The assistance scheme of the developed system was defined as follows for the coordinated multijoint limb tasks:

(1) $$\begin{array}{cc} & Exo-neuro-musculo-skeletalassistance=\\ & \left\{\begin{array}{c} V_{EMG,TRI}∙Assistance_{elbow}\left(NMES+MusculoskeletonInflation\right),inelbowextension\\ V_{EMG,ECU-ED}∙\left[Assistance_{wrist}\left(NMES\right)+Assistance_{fingers}\left(NMES+MusculoskeletonInflation\right)\right],inwristextensionhandsopen\\ V_{EMG,FCR-FD}∙\left[Assistance_{wrist}\left(NMES\right)+Assistance_{fingers}\left(NMES+Musculoskeletondeflation\right)\right],inwristflexionhandclosed\\ V_{EMG,BIC}∙Assistance_{elbow}\left(NMES+MusculoskeletonDeflation\right),inelbowflexion \end{array}\right. \end{array}$$

where three times the standard deviation (SD) above the EMG baseline in the resting state was set as a threshold level in each motion phase. When the EMG level of a driving muscle or muscle union *m* reached a preset threshold, the value of $V_{EMG,m}$was 1 and assistance was simultaneously triggered from both the musculoskeleton and NMES to assist the extension or flexion of the related joint. When the EMG level did not reach the preset threshold, the value of $V_{EMG,m}$ was 0.

The parameter *Assistance_elbow_ (NMES + Musculoskeleton Inflation)* is the assistance provided during elbow extension, including the NMES (with a threshold pulse width to evoke visible elbow extension)^45^ applied to the TRI muscle and the mechanical extension torque provided to the elbow joint by the inflated elbow musculoskeleton. The parameter $Assistance_{wrist}\left(NMES\right)+Assistance_{fingers}\left(NMES+Musculoskeletoninflation\right)$ is the assistance provided during wrist extension with the hand open, including the NMES (with a threshold pulse width to evoke maximal wrist extension with full-finger extension)^45^ applied to the ECU-ED muscle union and the mechanical extension torque provided to the fingers by the inflated hand musculoskeleton.

The parameter $Assistance_{wrist}\left(NMES\right)+Assistance_{fingers}\left(NMES+Musculoskeletondeflation\right)$ is the assistance provided during wrist flexion with the hand closed, including the NMES (with a threshold pulse width to evoke maximal wrist flexion with full-finger flexion)^45^ applied to the FCR-FD muscle union, and the hand musculoskeleton could be deflated passively during the aforementioned assistance. Most stroke survivors could perform voluntary finger flexion, but most of them cannot extend their fingers.^46^ The residual voluntary effort from the finger flexors of the paretic limb would facilitate the release of the air from the musculoskeleton in deflation. The parameter $Assistance_{elbow}\left(NMES+MusculoskeletonDeflation\right)$ represents the assistance provided during elbow flexion, including the NMES (with a threshold pulse width to evoke visible elbow flexion)^45^ applied to the BIC muscle, and the elbow musculoskeleton could be deflated passively during the aforementioned assistance. Meanwhile, the residual voluntary effort from the elbow flexors of the paretic limb would facilitate the release of the air from the musculoskeleton in deflation.

### Mechanical structure of the musculoskeletons

The elbow musculoskeleton, which had a length of 24 cm (the detailed dimensions are presented in Fig. 1b), was composed of one piece of pneumatic muscle (polyvinyl chloride [PVC] membrane, 1 mm thick) in the middle and an exoskeletal extension at each end. The musculoskeleton provided extension torque when the pneumatic muscle was inflated by the air compressor. A three-dimensional (3D)-printed plastic (photopolymer) extension (height: 4 cm, width: 6 cm, and thickness: 1 cm) was connected and sealed at each end of the pneumatic muscle. The connections were pressed with two aluminum plates and fastened using rivets. The elbow musculoskeleton was attached to the ventral side of the elbow, with its geometric center located at the joint on the paretic arm around which an elastic sleeve-like bracing (spandex) was wrapped. The musculoskeletons were integrated inside of an elastic bracing (spandex) with the purpose to achieve an average pressure applied to the skin surface from 1279 to 2860 Pa during the inflation and deflation of the pneumatic muscles for the needed mechanical assistance, as well as stable and comfortable wearing experience.^47,48^

The hand musculoskeleton (Fig. 1b) comprised five pneumatic finger muscles (PVC membrane, 1-mm thick), one for each digit (width of 1.6 cm for each pneumatic muscle; thumb length = 12.5 cm, index finger length = 17.5 cm, middle finger length = 18.5 cm, ring finger length = 18.0 cm, and little finger length = 14.5 cm), that converged to a 3D-printed exoskeletal connector (photopolymer) at the end of the musculoskeleton, which connected to the air compressor. Each pneumatic finger muscle generated extension torque for the fingers during inflation. The hand musculoskeleton was embedded in an elastic glove-like bracing (spandex) and fixed on the palm during hand opening or closing movements, with the exoskeletal connector located near the bottom of the palm.

The maximal inner pressures of the pneumatic muscles of both musculoskeletons were set at <100 kPa to maintain the stability of the musculoskeletons under repeated inflations and deflations. The proportions and lengths of the musculoskeletons were selected according to mean values of the upper limb anthropometrics for Asian adults.^49,50^

### Pressure/torque transmission of the musculoskeletons

The pressure/torque transmission properties of the musculoskeletons were quantified by determining the relationship between the inner pressure and extension torque of the musculoskeletons. The pressure/torque transmission rate was determined as the response time of each musculoskeleton for achieving a preset maximal inner pressure. The musculoskeletons for the elbow and hand were evaluated separately in this study.

#### Elbow module

**FIG. 3.**
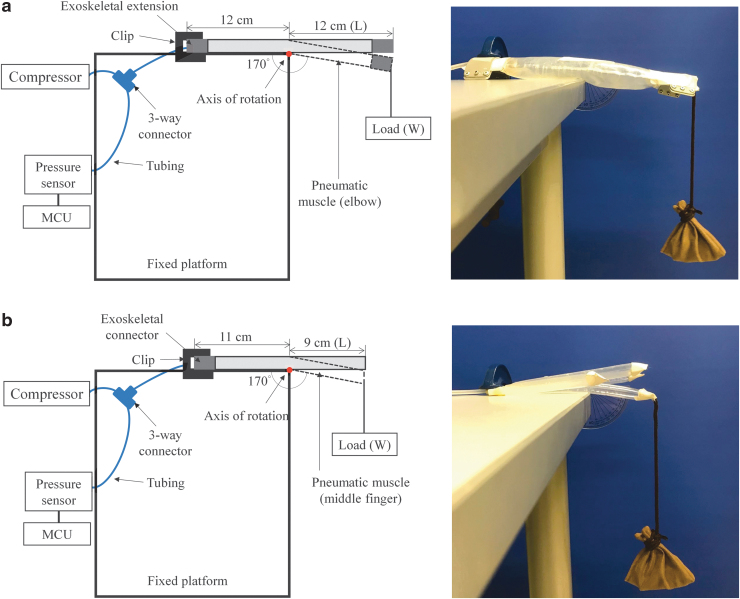
Experimental setup for the evaluation of the pressure/torque transmission properties of the musculoskeleton for the **(a)** elbow and **(b)** hand. MCU, microcontroller unit.

The experimental setup for measuring the pressure/torque transmission of the elbow musculoskeleton during extension is depicted in Figure 3a. One end of the skeletal extension was fixed on a platform, with half the length of the musculoskeleton falling outside the platform. The configuration in Figure 3a was used to evaluate the extension torque provided to the elbow joint, when the elbow musculoskeleton was attached to the elbow and extended around the center located at the elbow joint.

The musculoskeleton was inflated by the compressor with the fully opened valve till the inner pressure reached 95 kPa, at which the elbow musculoskeleton was fully extended to 180°. Then, a weighed loading (sandbag) was hanged to the unfixed end of the exoskeletal extensions. The total hanging weight increased until the musculoskeleton flexed at the joint position with an angle of 170° because most chronic stroke patients with muscle spasticity can reach 170° of the elbow joint passively.^51^ When adding the load, the inner pressure was maintained at <100 kPa. The change in the joint angle was measured using a protractor whose midpoint was aligned with the rotation center of the musculoskeleton. The total weight of loading and the corresponding reading of the inner pressure were recorded. The inner pressure of the musculoskeleton was then decreased in steps of 5 kPa with an error within 1 kPa. The measuring scale was set from 5 to 95 kPa without loading in this study because a minimum inner pressure of 5 kPa was required to achieve a joint angle of 180° for the elbow musculoskeleton under free loading. The measurement was repeated three times for each scaling step. A similar evaluation method was adopted in a study on a pneumatic elbow sleeve.^52^ The produced output torque related to each measured pressure was calculated as follows:

(2) $$Torque=L\cdot cos10^{\circ }\cdot W$$

whereL is the length between the axis of rotation and the endpoint of exoskeletal extension with loading and *W* is the weight of the loading.

The response time of the elbow musculoskeleton during inflation was recorded under free loading. The response time was used to measure the baseline performance of the elbow module before the module was used to provide joint assistance to humans. The musculoskeleton was fixed on a platform with the configuration depicted in Figure 3a. The musculoskeleton was then inflated from an inner pressure of 0 to <100 kPa without external loading with the fully opened valve. The measurements were repeated thrice.

#### Hand module

The pressure/torque transmission of the hand module was assessed using the middle finger as a representative finger for the measurement of the pressure/torque relationship (Fig. 3b). The middle finger was considered the representative finger because it has the longest extended length and the largest air volume among the fingers in the evaluation of the finger extension torque of the musculoskeleton. The configuration in Figure 3b was used to evaluate the extension torque provided to the metacarpophalangeal (MCP) joint of the middle finger when the musculoskeleton was attached to the palm and extended around the center located near the MCP joint, which was the primary joint when the hand was open.^53^ The exoskeletal connector of the hand module was fixed on the platform with the pneumatic middle finger, which was present in the palm area inside the platform. The length of the middle finger musculoskeleton inside the platform was 11 cm, which represents the mean length of the wrist joint and MCP joint of the middle finger on the palmar side of Asian adults.^50^

The hand musculoskeleton, including all the pneumatic fingers, was inflated and deflated simultaneously during the experiment. The procedures for the pressure/torque measurement performed on the middle finger musculoskeleton and the response time measurement performed on the hand musculoskeleton having the configuration depicted in Figure 3b were similar to those used to assess the pressure/torque relationship and response time of the elbow musculoskeleton.

### Evaluation of joint assistance by the EMG-driven exoneuromusculoskeleton

The assistive capability of the EMG-driven exoneuromusculoskeleton was evaluated on patients with chronic stroke by using four assistance schemes, as presented in Table 1, to understand the different assistance contributions of NMES and the musculoskeleton to the upper limb movements. After obtaining ethical approval from the Human Subjects Ethics Subcommittee of the Hong Kong Polytechnic University, 10 participants with chronic stroke were recruited for the evaluation. The demographic data of the participants in the evaluation are presented in Table 2. Written informed consents were obtained from all the recruited participants in this study. The inclusion criteria were as follows: (1) at least 1 year after the onset of a singular and unilateral brain lesion due to stroke; (2) the spasticity at the elbow, wrist, and fingers was ≤3 as measured by the Modified Ashworth Scale (MAS)^54^; (3) motor impairments in the affected upper limb range from severe to moderate according to the Fugl-Meyer Assessment (FMA; 15 < FMA <45, with a maximal score of 66 for the upper limb)^55^; (4) presence of no visual deficit and the ability to understand and follow simple instructions, as assessed by the Mini-Mental State Examination (MMSE >21)^56^; (5) presence of detectable voluntary EMG signals from the driving muscle on the affected side (three times the SD above the EMG baseline); and (6) presence of passive ROM for the wrist from 45° extension to 60° flexion, presence of passive ROM for the elbow from 30° to 170°, and ability of the MCP finger joints to be passively extended to 170°.

**Table 1.** Notations for the Different Assistance Schemes of the Exoneuromusculoskeleton

Notation of assistance schemes	Description
N0M0	No assistance from either the musculoskeleton or the NMES
N1M0	Assistance from the NMES only
N0M1	Assistance from the musculoskeleton only
N1M1	Assistance from both the musculoskeleton and the NMES

NMES, neuromuscular electrical stimulation.

**Table 2.** Demographic Characteristics of the Participants Recruited for the Range of Motion Measurements (*n* = 10)

Subjects no.	GenderFemale/male	Stroke typesHemorrhagic/ischemic	Side of hemiparesisLeft/right	Age (years)Mean ± SD	Years after onset of strokeMean ± SD	FMAMean ± SD	MAS elbowMean ± SD	MAS wristMean ± SD	MAS fingerMean ± SD
10	4/6	4/6	6/4	64.1 ± 5.89	5.60 ± 3.98	37.2 ± 11.6	1.46 ± 0.39	1.54 ± 1.19	1.44 ± 0.91

FMA, Fugl-Meyer Assessment; MAS, Modified Ashworth Scale; SD, standard deviation.

#### Evaluation

The evaluation comprised three sessions for the measurement of assistive performance of the developed system for the elbow, wrist, and finger joints. The performance of each joint was evaluated according to the ROM achieved under different assistance schemes (Table 1).

#### Elbow and wrist sessions

The ROMs related to the elbow and wrist joints were measured separately through motion capturing. In total, 25 spherical reflective markers (12 mm diameter for each) were attached to the skin with double-sided tape according to the upper limb model of the BodyBuilder model (Vicon Motion Systems, Oxford, United Kingdom).^57^ The marker positions were captured through an eight-camera motion system (Vicon Motion Systems), with a sampling frequency of 250 Hz. A Vicon Workstation (Vicon Motion Systems) with 3D reconstruction software (Vicon Nexus and BodyBuilder, Oxford, United Kingdom) was used to anatomically label, filter, and apply the upper limb model.^57–59^ The dynamic joint angles during motion were thus obtained during all trials.^60^ The ROMs of the target joints in the extension phase were investigated because most patients with chronic stroke experience impairment in joint extension in the upper limb rather than in flexion.^46^ Most of the poor limb performance (e.g., open hand to grasp) was related to an inability to activate extensor muscles on the upper limb.^61^

**FIG. 4.**
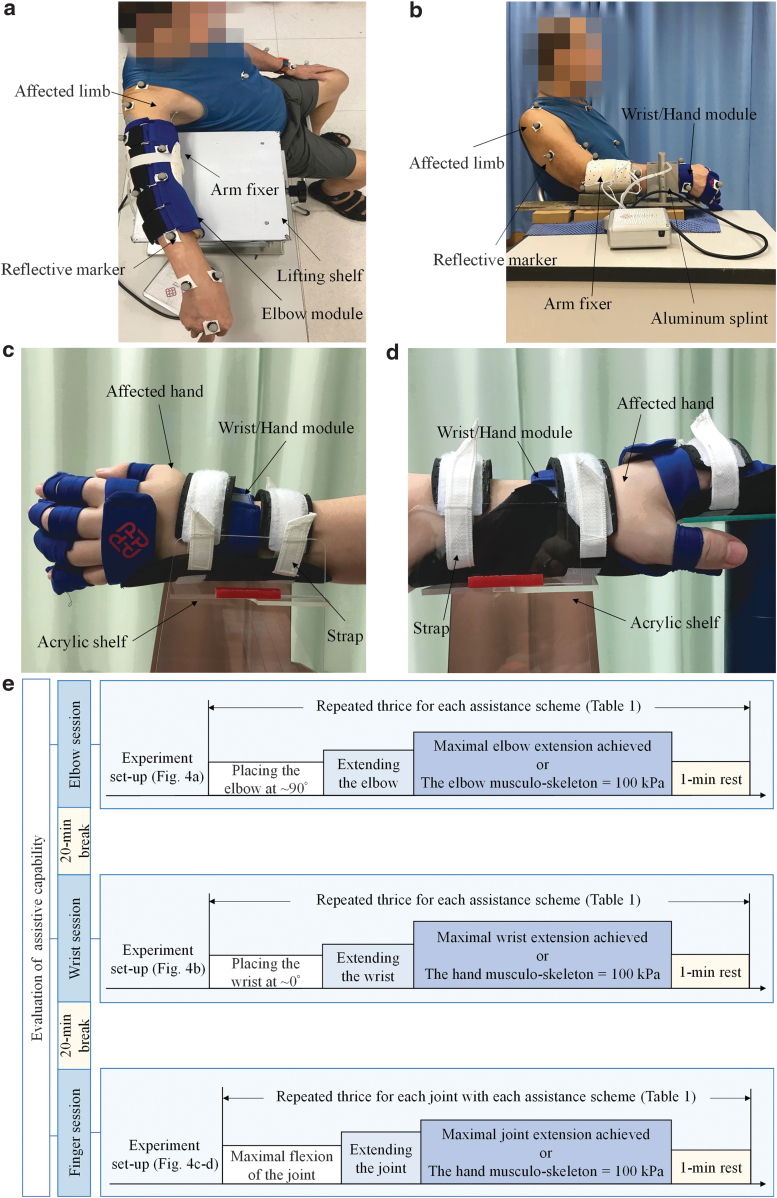
Seating configuration during the evaluations in the **(a)** elbow and **(b)** wrist sessions as well as the experiment setup of finger joint goniometric measurements for the **(c)** index, middle, ring, and little fingers and **(d)** thumb, and **(e)** the evaluation protocol presented with time line.

In the elbow session, the participants wore the elbow module on the affected limb and sat on a 45-cm-high straight-back chair in front of a 72-cm-high table (Fig. 4a). The tested arm was positioned using an upper arm fixer on a lifting shelf placed near the table edge. In the initial position, the forearm was pronated and the shoulder was positioned at 80° vertical abduction with ∼10° flexion. The participants' unaffected hand rested on their thigh. The participants were required to perform a task that involved placing their elbow at ∼90° initially and then extending their elbow to their maximal angle. The participants were instructed to complete the task at their natural speed after the experiment operator provided them an audio starting signal. The trial was completed when they reported that they had achieved their maximal elbow extension or when the inner pressure of the elbow musculoskeleton reached 100 kPa. All the participants reported the completion of the trial before the inner pressure reached 100 kPa within 25 s. The recorded trial lengths were sorted in ascending order from 0 to 21 s and used for comparing the response time in the elbow session because a stable value (defined as <1% change in the maximum value) was achieved within 21 s in all the trials.

In the wrist session, the participants wore the wrist/hand module on the affected limb and sat on the same chair as in the elbow session (Fig. 4b). The table used in the wrist session was also the same as that used in the elbow session. The tested arm was positioned using a forearm fixer and a splint that attached to the table edge. The shoulder was positioned at 20° lateral rotation with ∼30° vertical abduction. The elbow was positioned with a joint angle of 140°, and the unaffected hand rested on the thigh. The participants were required to conduct a wrist task that involved placing the wrist at ∼0° initially (i.e., the neural position for extension or flexion) and then extending the wrist to their maximal angle. After the experiment operator provided an audio starting signal, the participants were required to perform the task at their natural speed. The trial was completed when they reported that the maximal wrist extension was reached or when the inner pressure of the hand musculoskeleton reached 100 kPa. Although the wrist movement was only supported by NMES in this study, four assistance schemes (Table 1) were used in the evaluation of the ROM of the wrist joint because the assistance provided by the exoneuromusculoskeleton for the wrist was triggered in conjunction with that for the fingers in the same motion phases (i.e., wrist extension with the hand open and wrist flexion with the hand closed) during limb practice. The developed system assisted coordinated movements between the wrist and the fingers for the participants in this study. Moreover, a study reported that the wrist ROM can be affected by finger positions.^62^

All the participants reported the completion of the trials within 16 s. The recorded trial lengths were sorted in ascending order from 0 to 13 s and used for comparing the response time in the wrist session because all trials reached a stable value in <13 s.

The ROM of each joint in the task was measured by comparing the final and initial joint angles. The participants performed the task three times with each assistance scheme (Table 1) in random order. Thus, each participant performed 12 trials in each session. A 1-min rest period was provided between two consecutive trials to prevent fatigue.

#### Finger session

The finger ROMs were obtained through manual goniometric measurements because the main impairment in the hand for patients with chronic stroke is hand opening, during which fingers flex passively due to spasticity in the finger flexors.^46^ Attaching markers on the spastic finger joints of the recruited participants was not feasible.

In the finger session, the participants wore the wrist/hand module on the affected limb and sat on a 45-cm-high straight-back chair in front of a 72-cm-high table. The tested hand was fixed 12 cm from the table edge in the midline of an acrylic shelf with straps. The participants' other hand rested on their thigh. The wrist was positioned at ∼0° during the evaluation of the MCP, proximal interphalangeal (PIP), and distal interphalangeal (DIP) joints of the index, middle, ring, and little fingers (Fig. 4c). When conducting measurements on the MCP and DIP joints of the thumb, the hand was pronated and the wrist was positioned at ∼0° (Fig. 4d). The aforementioned configurations were used to minimize the gravity effect on the finger movements.

During the measurements, the participants were required to perform their maximal flexion and extension for each joint at their natural speed in a trial. A trial was initiated when the participants reported that they had reached their maximal flexion and was completed when the participants reported that they had achieved their maximal extension or when the inner pressure of the hand musculoskeleton reached 100 kPa. A video camera was used during the measurement, and videos were recorded at a frame rate of 30 fps to confirm the movement timing in each trial. All the participants reported the completion of the trials within 12 s before the inner pressure reached 100 kPa. The finger joint angles were obtained manually by placing the axis of a finger goniometer on the dorsal part of each joint.^63^ Each joint was measured thrice with the four assistance schemes in random order. Thus, 12 trials were performed for each joint. A 1-min rest period was provided between two consecutive trials to prevent fatigue. The ROM of each measured finger joint was recorded by measuring the angles between the beginning position (i.e., at a maximal flexion angle) and the final position (i.e., at a maximal extension angle) in a trial. In addition to the ROM of each measured finger joint, the ROM of each finger (SUM_ROM) was defined as the sum of the ROMs of its measured joints (i.e., the MCP, PIP, and DIP joints for the index, middle, ring, and little fingers and the MCP and DIP joints for the thumb).

Each participant was required to complete all the elbow, wrist, and finger sessions on the same day. A 20-min break was provided between two consecutive sessions to avoid fatigue. Figure 4e illustrates the evaluation protocol presented with the time line.

### Self-help upper limb training assisted by the EMG-driven exoneuromusculoskeleton

A pilot clinical trial with a single-group design was conducted to investigate the feasibility and rehabilitation effects of self-help upper limb training assisted with the EMG-driven exoneuromusculoskeleton. A total of 15 participants with chronic stroke who met the same inclusion criteria as in the aforementioned evaluations were recruited in the pilot trial after obtaining ethical approval from the Human Subjects Ethics Subcommittee of the Hong Kong Polytechnic University. The demographic data of the participants in the pilot clinical trial are presented in Table 3. Written consent was obtained from each participant before clinical trial commencement.

**Table 3.** Demographic Characteristics of the Participants Recruited for the Electromyography-Driven Exoneuromusculoskeleton-Assisted Self-Help Upper Limb Training (*n* = 15)

Subjects No.	Gender	Stroke types	Side of hemiparesis	Age (years)	Years after onset of stroke
	Female/male	Hemorrhagic/ischemic	Left/right	Mean ± SD	Mean ± SD
15	5/10	8/7	7/8	59.8 ± 8.20	6.07 ± 4.28

#### Training protocol

All the participants received self-help upper limb training assisted with the EMG-driven exoneuromusculoskeleton. The training comprised 20 sessions, with a training intensity of 3–5 sessions/week, within seven consecutive weeks.

Before the training, a tutorial session was provided to each participant on the device operation, electrode attachment (the electrode positions were marked on the skin by an experiment operator), wearing skills, and the training protocol. In the first three training sessions, professional assistance was provided in a rehabilitation laboratory at varying levels. The levels of support can be described as follows: (1) the operator supported the participants during the setup and supervised the entire training process in the first session (fully assisted session); (2) the participants mainly completed the session by themselves, with minimum assistance from the operator in the second session (semiassisted session); and (3) the participants completed the third session independently but with close observation by the operator (independent-with-observation session). Additional semiassisted sessions were offered to participants who were not ready for the independent-with-observation session; however, no additional semiassisted session was required by the participants in this study. In the remaining training sessions (i.e., the 4th to 20th sessions), the participants performed the required tasks independently in the laboratory without close supervision of the operator. The operator provided help if required (e.g., if an electrode lead was broken).

**FIG. 5.**
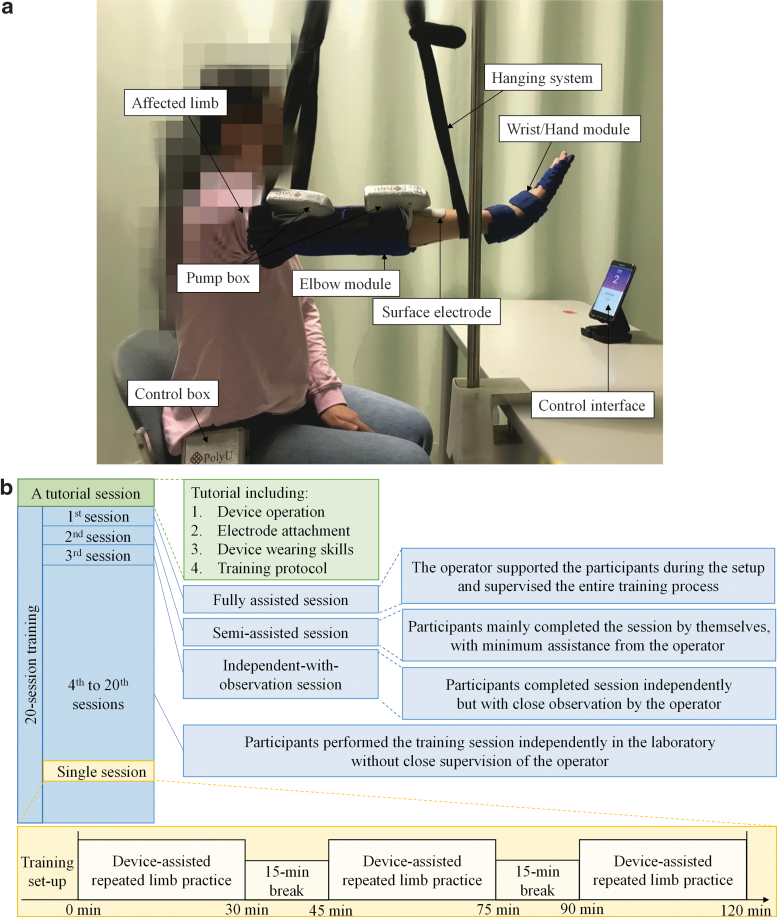
**(a)** Experimental training setup of the EMG-driven exoneuromusculoskeleton in the laboratory, and **(b)** the training protocol presented with time line.

In each training session, the participants were seated at a table to maintain a vertical distance of 30–40 cm between the table surface and their shoulder. During the task, the participants' paretic upper limb with the wearable modules was lifted up to 80° vertical abduction of the shoulder with a hanging system (Fig. 5a). A smartphone was positioned on the table and placed in front of the participant with a horizontal distance of 60 cm. The participants were instructed through a visual indication on the mobile screen to perform device-assisted and repeated limb motions, namely (1) elbow extension, (2) wrist extension with the hand open, (3) wrist flexion with the hand closed, and (4) elbow flexion, at their natural speed (for totally 90 min in each session). A 15-min break was provided between two consecutive 30-min practice to prevent muscle fatigue. Figure 5b shows the training protocol presented with time line.

#### Clinical assessments

In this study, the training effects were assessed through clinical assessments of the FMA that the full score is 66 for the upper limb assessment and has been subscaled into shoulder/elbow (42/66) and wrist/hand (24/66),^64^ the Action Research Arm Test (ARAT),^65^ the Wolf Motor Function Test,^66^ the Motor Functional Independence Measure,^67^ and the MAS^54^ at the elbow, wrist, and fingers. These clinical assessments were performed thrice in 2 weeks before the training for detection of the baseline stability. The aforementioned clinical assessments were also performed immediately after the last training session and 3 months after the training by a training-blinded assessor.

#### Cross-sessional evaluation through EMG

At the beginning of each training session, EMG recordings of the maximum voluntary contractions (MVCs) and a bare arm test (performed in previous studies^39,44^) were performed.

Each participant first received an MVC test^68^ for the following target muscle unions or muscles, that is, the ECU-ED, FCR-FD, TRI, and BIC. While conducting the MVC test on the ECU-ED and FCR-FD, participants were seated at a table and the paretic upper limb was placed on the table with the elbow joint extended to an angle of 130°, and the wrist was held by an experimental operator positioned around its neutral position. The finger positions were set by the operator to obtain an angle around 150° at the MCP joints of the index, middle, ring, and little fingers. During the isometric maximum voluntary extension (IMVE) of the wrist and the four fingers, the ECU-ED EMG signals were recorded; and during the isometric maximum voluntary flexion (IMVF) of the wrist and the four fingers, the FCR-FD EMG signals were captured. During the MVC test on the TRI and BIC, the paretic upper limb was positioned with the shoulder abducted at 70° and the elbow flexed at 90°. During the IMVE and IMVF of the elbow, the TRI and BIC EMG signals were recorded, respectively. The MVC test on each target muscle, or muscle union, was repeated twice and the contraction was maintained for 5 s. The variation of maximum EMG amplitude in the two repetitions was required to be within 10%, otherwise, the MVC test would be repeated. The largest EMG amplitude was then selected as the EMG amplitude of MVC for the target muscle union or muscle. A 2-min break was provided between two consecutive contractions to avoid muscle fatigue.

The bare arm test comprised horizontal arm reaching, hand grasping, and withdrawing motions, which were similar to the limb practice motions during the training task. The participants were required to use their paretic limbs (without assistance from the system) to repeat the test three times at their natural speed.

EMG electrodes (2 × 3 cm^2^, Blue Sensor N; Ambu, Inc.) were attached to the skin surface of the aforementioned target muscle unions and muscles (the configuration specified in a previous study^40,69^ was used). The collected EMG signals were amplified with a gain of 1000 (amplifier: INA 333; Texas Instruments, Inc.), band-pass filtered from 10 to 500 Hz, and then sampled with 1000 Hz for digitization for offline processing.^69,70^ Two EMG parameters were calculated for quantitative session-by-session monitoring of the evolution of the muscle activation and coordination patterns: (1) the normalized EMG activation level of each target muscle and (2) the normalized EMG cocontraction index (CI) between muscle pairs.^71,72^ The EMG activation level of a muscle was calculated as follows:

(3) $$\bar{EMG}=\frac{1}{T}\int_{0}^{T}EMG_{i}\left(t\right)dt$$

where $\bar{EMG}$ refers to the average EMG envelope value of muscle *i*, $EMG_{i}\left(t\right)$ is the EMG envelope signal obtained after normalization with respect to the EMG MVC value of the muscle, and *T* is the length of the signal.

The CI between a pair of muscles can be expressed as follows:

(4) $$CI=\frac{1}{T}\int_{0}^{T}A_{ij}\left(t\right)dt$$

where A_ij_(t) is the overlapping activity of EMG linear envelopes for muscles *i* and *j* and *T* is the length of the signal. An increase in the CI value represents increased cocontraction of a muscle pair (broadened overlapping area), and a decrease in the CI value indicates decreased cocontraction of a muscle pair (reduced overlapping area). To obtain the tendency of the EMG parameters of an individual with normalized values (varying from 0 to 1) and to minimize the variations among different participants, a further normalization was applied to the aforementioned EMG parameters of individual participants with respect to the maximal and minimal values of the participants across the 20 training sessions.^39,69^

### Statistics

The normality tests on the ROMs, clinical scores, and EMG data were evaluated using the Lilliefors method with a significance level of 0.05.^73^ The ROMs of the wrist and finger joints exhibited significance in the normality test (*p* < 0.05), and the ROMs of the elbow, the clinical score, and the EMG data exhibited nonsignificant probabilities (*p* > 0.05). Kruskal–Wallis one-way analysis of variance (ANOVA) with the Bonferroni *post hoc* test was used to evaluate the differences in the ROMs of the wrist and finger joints with the four assistance schemes. One-way ANOVA with the Bonferroni *post hoc* test was used to detect the differences in the ROMs of the elbow with the four assistance schemes, and to evaluate the differences in the clinical assessments across different time points (three pretraining assessments, a post-training assessment, and a 3-month follow-up assessment) and the EMG parameters (i.e., the normalized EMG activation levels and normalized CIs) across the 20 training sessions. The statistically significant level was set as 0.05 in this study. The significance levels at 0.01 and 0.001 are also indicated.

## Results

### Pressure/torque transmission of the musculoskeletons

#### Elbow module

**FIG. 6.**
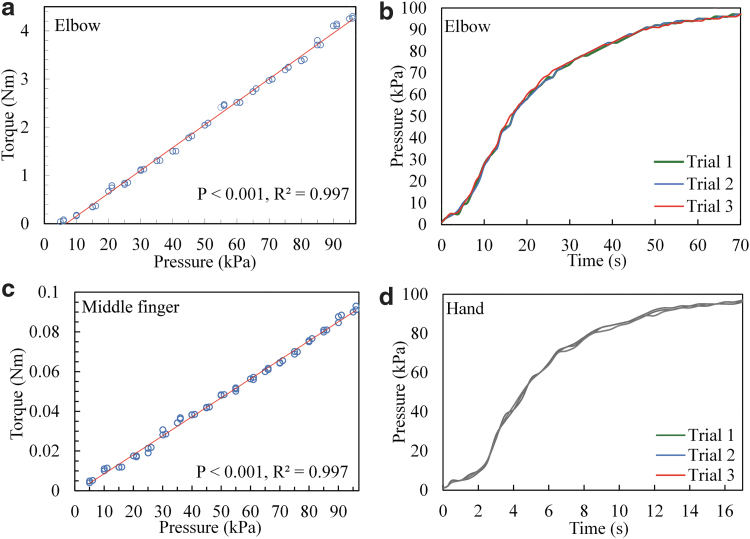
**(a)** Pressure/torque relationship and **(b)** response time of the inner pressure of the elbow musculoskeleton during inflation with a fully opened valve; **(c)** pressure/torque relationship of the musculoskeleton for the MCP joint of the middle finger; and **(d)** response time of the inner pressure of the hand musculoskeleton during inflation with a fully opened valve. MCP, metacarpophalangeal.

The experimental result for the pressure/torque relationship during the inflation of the elbow musculoskeleton is depicted in Figure 6a. A significant linear relationship was found between the pressure and the torque for the elbow musculoskeleton (*p* ≤ 0.001, *R*^2^ = 0.997). The measured maximum extension torque was 4.3 Nm, which corresponded to an inner pressure of 96 kPa during inflation. Moreover, the torque-to-weight ratio was 27.2 Nm/kg (because the weight of the elbow module was 158 g). The pressure/time relationship for the elbow musculoskeleton is depicted in Figure 6b. During inflation, the inner pressure of the elbow musculoskeleton reached ≥96 kPa in ≤66 s under free loading.

#### Hand module

A significant linear pressure/torque relationship was detected when the musculoskeleton was used on the MCP joint of the middle finger (*p* ≤ 0.001, *R*^2^ = 0.997; Fig. 6c). When the maximal measured inner pressure reached 96 kPa, the corresponding extension torque of the MCP joint of the middle finger was 0.093 Nm. The torque-to-weight ratio was 9.3 Nm/kg, and the total weight of the middle finger was 10 g. The pressure/time relationship for the hand musculoskeleton is depicted in Figure 6d. During inflation, the inner pressure of the hand musculoskeleton reached ≥96 kPa within 17 s under free loading.

### Evaluation of joint assistance by the EMG-driven exoneuromusculoskeleton

**FIG. 7.**
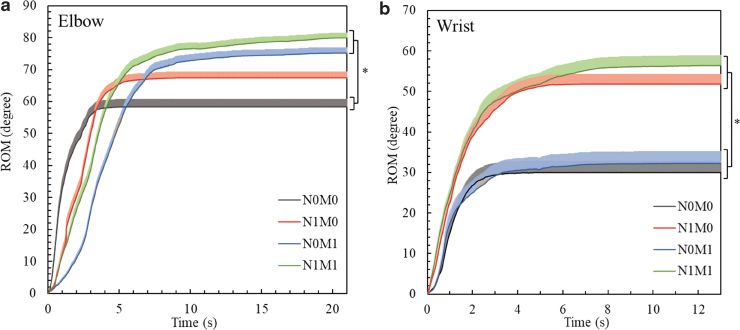
Comparison of the dynamic ROM values, which are represented in terms of their means (shaded areas indicate half an SE), at the **(a)** elbow and **(b)** wrist joints under the different assistance schemes. Significant differences (*p* ≤ 0.05) with respect to the assistance scheme are indicated by “*.” ROM, range of motion; SE, standard error.

Figure 7a and b depicts the ROM variations recorded with different assistance schemes in the evaluation of the elbow and wrist joints, respectively. As shown in Figure 7a, the elbow ROM was significantly larger with the assistance from the musculoskeleton (N0M1 and N1M1) than without any assistance from the system (N0M0) (*p* = 0.002, effect size [EF] = 0.123, *F* = 5.42, one-way ANOVA with the Bonferroni *post hoc* test). With no assistance from the system (N0M0), the elbow ROM achieved its steady state (defined as the ROM value >95% of the stable value) in ∼3 s. With only NMES assistance (N1M0) from the system, the elbow ROM reached its steady state in ∼5 s. With mechanical assistance (N0M1 and N1M1) from the system, both elbow ROM values achieved their steady state in ∼9 s. As shown in Figure 7b, the wrist ROM was significantly larger when the system provided NMES assistance (N1M0 and N1M1) than when the system did not provide NMES support (N0M0 and N0M1) (*p* ≤ 0.001, Kruskal–Wallis one-way ANOVA with the Bonferroni *post hoc* test). With no assistance from the system (N0M0), the wrist ROM achieved its steady state in ∼2.5 s. With only NMES assistance (N1M0) and only mechanical assistance (N0M1) from the system, the corresponding wrist ROMs achieved their steady state in ∼4 s. With both NMES and mechanical assistance (N1M1) provided by the system, the wrist ROM achieved its steady state in ∼6 s.

The elbow and wrist ROM values measured in this study (i.e., means and 95% confidence intervals of the related joints as well as the one-way ANOVA probabilities with the EF or Kruskal–Wallis one-way ANOVA probabilities for the evaluation with respect to the different assistance schemes) are presented in Table 4.

**Table 4.** Means and 95% Confidence Intervals for Each Measurement of the Elbow, Wrist, and Finger Joints, as Well as the Probabilities of the Statistical Analyses

ROM	N0M0	N1M0	N0M1	N1M1	One-way ANOVA	
	Mean (95% confidence interval)				p-Value (partial η^2^)	F-value
Elbow joint	58.3 (48.0–68.7)	67.4 (59.2–75.6)	75.2 (67.6–82.7)	80.0 (73.3–86.7)	0.002^**^ (0.123)	5.42
*ROM*	*N0M0*	*N1M0*	*N0M1*	*N1M1*	*Kruskal–Wallis one-way ANOVA*	
	*Mean (95% confidence interval)*				p*-Value*	
Wrist joint	29.9 (17.5–42.4)	51.8 (41.6–62.1)	32.2 (19.5–45.0)	56.4 (45.6–67.3)	0.000^***^	
Thumb						
SUM_ROM	77.7 (55.2–100)	94.0 (71.8–116)	124 (112–136)	124 (112–136)	0.008^**^	
MCP joint	31.8 (21.0–42.6)	36.8 (24.7–49.0)	45.7 (36.2–55.1)	45.7 (36.2–55.1)	0.146	
DIP joint	45.8 (33.4–58.2)	57.2 (46.3–68.0)	78.3 (73.8–82.9)	78.3 (73.8–82.9)	0.000^***^	
Index finger						
SUM_ROM	90.2 (58.7–122)	186 (168–204)	205 (195–215)	210 (201–220)	0.000^***^	
MCP joint	30.8 (21.4–40.2)	68.5 (61.5–75.5)	70.7 (63.2–76.8)	71.7 (65.0–78.3)	0.000^***^	
PIP joint	39.0 (25.0–53.0)	72.5 (63.7–81.3)	89.5 (85.6–93.4)	89.5 (85.6–93.4)	0.000^***^	
DIP joint	20.3 (11.0–29.7)	44.8 (37.5–52.2)	45.3 (38.9–51.7)	48.8 (44.0–53.7)	0.000^***^	
Middle finger						
SUM_ROM	92.3 (62.2–123)	198 (181–216)	212 (204–220)	215 (208–223)	0.000^***^	
MCP joint	26.7 (17.3–36.1)	73.0 (67.5–78.5)	69.8 (63.8–75.9)	73.0 (67.5–78.5)	0.000^***^	
PIP joint	42.7 (27.8–57.6)	80.2 (70.2–90.1)	91.2 (86.7–95.6)	91.2 (86.7–95.6)	0.000^***^	
DIP joint	23.0 (14.7–31.3)	45.2 (38.8–51.5)	50.7 (45.9–55.4)	51.2 (46.3–56.0)	0.000^***^	
Ring finger						
SUM_ROM	89.3 (60.8–118)	181 (162–201)	200 (185–214)	200 (186–215)	0.000^***^	
MCP joint	33.7 (25.0–42.3)	72.0 (66.5–77.5)	71.5 (66.1–76.9)	72.0 (66.5–77.5)	0.000^***^	
PIP joint	35.8 (18.7–53.0)	73.2 (58.4–87.9)	86.3 (74.5–98.1)	86.3 (74.5–98.1)	0.001^***^	
DIP joint	19.8 (12.7–26.9)	36.2 (27.4–44.9)	41.8 (32.9–50.8)	41.8 (32.9–50.8)	0.001^***^	
Little finger						
SUM_ROM	102 (70.1–133)	195 (174–215)	218 (205–231)	219 (206–232)	0.000^***^	
MCP joint	33.2 (24.3–42.0)	68.3 (58.4–78.3)	67.3 (57.5–77.1)	68.3 (58.4–78.3)	0.000^***^	
PIP joint	38.7 (22.3–55.1)	74.0 (63.2–84.8)	90.7 (86.4–95.0)	90.7 (86.4–95.0)	0.000^***^	
DIP joint	29.8 (20.9–38.8)	52.3 (44.6–60.1)	60.0 (55.0–65.0)	60.0 (55.0–65.0)	0.000^***^	

Differences with statistical significance are denoted using the notation “^*^.” The significant levels are indicated as ^**^ for *p* ≤ 0.01 and ^***^ for *p* ≤ 0.001.

ANOVA, analysis of variance; DIP, distal interphalangeal; MCP, metacarpophalangeal; PIP, proximal interphalangeal; ROM, range of motion; SUM_ROM, summation of the measured joints of one finger.

**FIG. 8.**
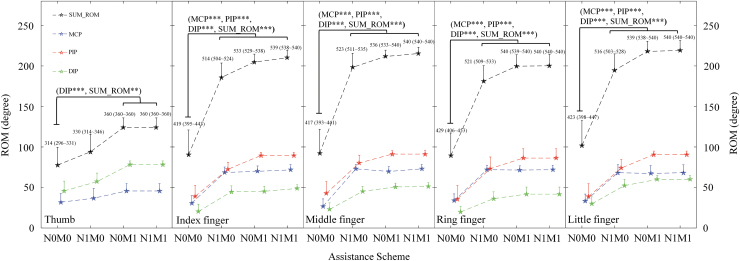
Comparison of the ROM values of the finger joints, which are represented in terms of their mean ± twice the SE (error bar), under different assistance schemes. Significant levels are indicated by ** for *p* ≤ 0.01 and *** for *p* ≤ 0.001. The total joint position of each finger is defined as the sum of the final position of each measured joint of the finger after hand opening (indicated with the means and 95% confidence intervals). DIP, distal interphalangeal; PIP, proximal interphalangeal; SUM_ROM, summation of the measured joints of one finger.

Figure 8 shows the ROM values recorded with different assistance schemes in the evaluation of the finger joints. The ROM values of the finger joints varied differently with the four assistance schemes. The SUM_ROM value of the thumb (*p* ≤ 0.01, Kruskal–Wallis one-way ANOVA with the Bonferroni *post hoc* test) and the ROM of the DIP joint of the thumb (*p* ≤ 0.001, Kruskal–Wallis one-way ANOVA with the Bonferroni *post hoc* test) were significantly higher when mechanical assistance was provided (N0M1 and N1M1) than when no assistance was provided from the system (N0M0). The SUM_ROM values of the index, middle, ring, and little fingers, as well as the ROMs of the MCP, PIP, and DIP joints of the index, middle, ring, and little fingers, were significantly higher when assistance was provided (N1M0, N0M1, and N1M1) than when no assistance was provided (N0M0) (*p* ≤ 0.001, Kruskal–Wallis one-way ANOVA with the Bonferroni *post hoc* test). The ROM values measured in this study for the finger joints (i.e., means and 95% confidence intervals of each joint as well as the Kruskal–Wallis one-way ANOVA probabilities for the evaluation with respect to the different assistance schemes) are listed in Table 4.

### Training effects

All the recruited participants (*n* = 15) completed self-help upper limb training assisted with the EMG-driven exoneuromusculoskeleton. The participants could wear and take off the developed system, and perform the training tasks independently (i.e., without close supervision and assistance from the operator) in the final 17 training sessions. The most frequently reported problem by the participants was broken leads during wearing and taking off the system, which was solved by on-site soldering or replacing the leads.

#### Clinical assessments

**FIG. 9.**
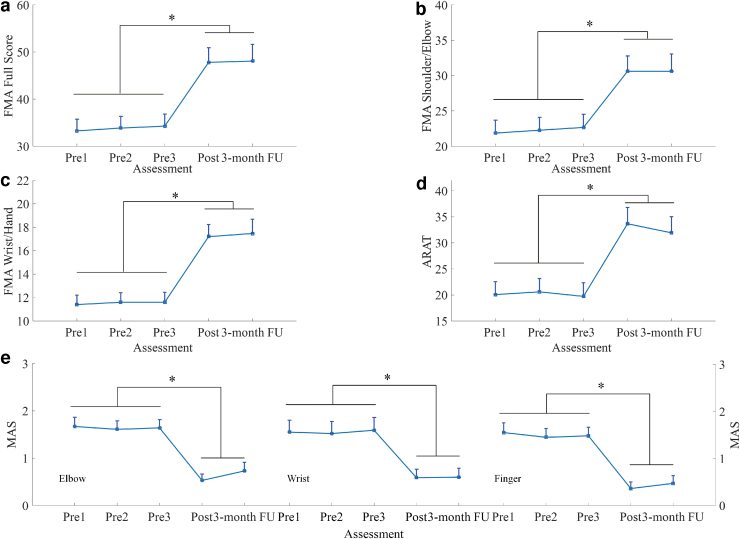
Clinical scores measured before, immediately after, and 3 months after the training: **(a)** FMA full scores, **(b)** FMA shoulder/elbow scores, **(c)** FMA wrist/hand scores, **(d)** ARAT scores, and **(e)** MAS scores at the elbow, wrist, and fingers. The clinical scores are presented as mean ± twice the SE (error bar) in each evaluation session. The significant difference is indicated by “*” (*p* ≤ 0.05). MAS, Modified Ashworth Scale.

Motor improvements measured by clinical scores (i.e., the FMA, ARAT, and MAS scores) are summarized in Figure 9. Significant increases were observed in the FMA full score (Fig. 9a; *p* ≤ 0.001, EF = 0.293, *F* = 7.27, one-way ANOVA with the Bonferroni *post hoc* test), FMA shoulder/elbow score (Fig. 9b; *p* ≤ 0.001, EF = 0.222, *F* = 5.00, one-way ANOVA with the Bonferroni *post hoc* test), and FMA wrist/hand score (Fig. 9c; *p* ≤ 0.001, EF = 0.386, *F* = 11.0, one-way ANOVA with the Bonferroni *post hoc* test) after the training, and these increases were maintained after 3 months. As depicted in Figure 9d, the ARAT score significantly increased after the training, and this increase was maintained for 3 months (*p* ≤ 0.001, EF = 0.262, *F* = 6.23, one-way ANOVA with the Bonferroni *post hoc* test). As shown in Figure 9e, the MAS scores at the elbow significantly declined after training, and this decline was maintained for 3 months (*p* ≤ 0.001, EF = 0.366, *F* = 10.1, one-way ANOVA with the Bonferroni *post hoc* test). Significant decreases were observed in the MAS scores at the wrist (*p* ≤ 0.001, EF = 0.229, *F* = 5.21, one-way ANOVA with the Bonferroni *post hoc* test) and fingers (*p* ≤ 0.001, EF = 0.391, *F* = 11.2, one-way ANOVA with the Bonferroni *post hoc* test) after the training, and these decreases were maintained after 3 months. Table 5 lists all the clinical scores measured in this study (i.e., means and 95% confidence intervals of each clinical assessment as well as the one-way ANOVA probabilities with the EF for evaluation with respect to the assessment sessions).

**Table 5.** Means and 95% Confidence Intervals for Each Measurement in the Clinical Assessments, as Well as the Probabilities and Estimated Effect Sizes of the Statistical Analyses

Evaluation	Pre 1	Pre 2	Pre 3	Post	3-month FU	One-way ANOVA	
	Mean (95% Confidence interval)					*p*-Value (partial η^2^)	*F*-value
FMA							
Full score	33.3 (28.0–38.6)	33.9 (28.5–39.2)	34.3 (28.8–39.8)	47.8 (41.2–54.5)	48.1 (40.4–55.7)	0.000^***^ (0.293)	7.27
Wrist/hand	11.4 (9.66–13.1)	11.6 (9.85–13.4)	11.6 (9.77–13.4)	17.2 (15.0–19.4)	17.5 (14.9–20.1)	0.000^***^ (0.386)	11.0
Shoulder/elbow	21.9 (17.9–25.8)	22.3 (18.4–26.2)	22.7 (18.7–26.7)	30.6 (25.9–35.3)	30.6 (25.4–35.9)	0.001^***^ (0.222)	5.00
ARAT	20.1 (14.7–25.4)	20.6 (15.1–26.1)	19.7 (14.1–25.4)	33.7 (26.9–40.4)	31.9 (25.3–38.6)	0.000^***^ (0.262)	6.23
WMFT							
Score	44.5 (36.3–52.7)	43.7 (35.4–51.9)	44.9 (36.5–53.2)	55.1 (47.8–62.5)	52.7 (44.3–61.2)	0.159 (0.088)	1.71
Time	38.8 (25.2–52.5)	40.1 (26.8–53.4)	40.6 (26.6–54.5)	24.2 (14.4–34.0)	28.0 (16.4–39.7)	0.107 (0.101)	1.98
FIM	66.0 (65.6–66.4)	66.0 (65.6–66.4)	66.0 (65.6–66.4)	66.1 (65.7–66.5)	66.1 (65.7–66.5)	0.954 (0.009)	0.167
MAS							
Elbow	1.67 (1.24–2.09)	1.61 (1.23–2.00)	1.64 (1.26–2.02)	0.53 (0.25–0.82)	0.73 (0.34–1.12)	0.000^***^ (0.366)	10.1
Wrist	1.56 (1.02–2.10)	1.53 (0.98–2.08)	1.60 (1.02–2.18)	0.59 (0.20–0.97)	0.60 (0.19–1.01)	0.001^***^ (0.229)	5.2
Finger	1.55 (1.10–1.99)	1.45 (1.06–1.85)	1.48 (1.09–1.87)	0.36 (0.06–0.66)	0.47 (0.11–0.82)	0.000^***^ (0.390)	11.2

Differences with statistical significance are denoted by the notation “^*^.” Significant levels are indicated as ^***^ for *p* ≤ 0.001.

3-month FU, 3-month follow-up assessment; ARAT, Action Research Arm Test; FIM, Functional Independence Measurement; FMA, Fugl-Meyer Assessment; Post, post-training assessment; Pre 1, first pretraining assessment; Pre 2, second pretraining assessment; Pre 3, third pretraining assessment; WMFT, Wolf Motor Function Test.

#### EMG parameters

**FIG. 10.**
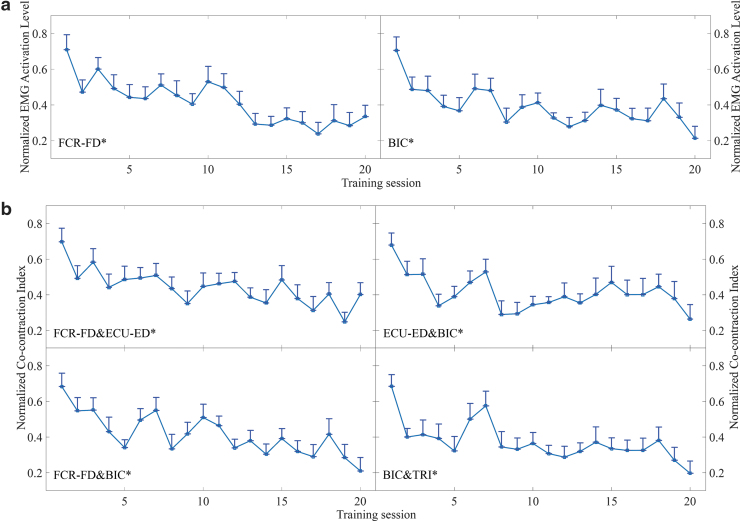
Variations in the EMG parameters recorded across the 20 training sessions: **(a)** the normalized EMG activation levels of the FCR-FD muscle union and BIC muscles during the bare hand evaluations and **(b)** the changes in the normalized CIs between the FCR-FD and ECU-ED muscle unions, the ECU-ED muscle union and the BIC muscles, the FCR-FD muscle union and BIC muscles, and the BIC and TRI muscle pair during the bare hand evaluations. The EMG parameter values are presented as mean ± twice the SE (error bar) for each session. The significant difference is indicated by “*” (*p* ≤ 0.05). CI, cocontraction index.

Figure 10 presents the EMG parameters (i.e., the normalized EMG activation level and normalized CI), which exhibited significant variations in the evaluations across the 20 training sessions. A significant decrease in the EMG activation level was observed for the FCR-FD muscle union (Fig. 10a; *p* ≤ 0.001, EF = 0.168, *F* = 2.98, one-way ANOVA with the Bonferroni *post hoc* test) and BIC muscle (Fig. 10a; *p* ≤ 0.001, EF = 0.138, *F* = 2.36, one-way ANOVA with the Bonferroni *post hoc* test). Figure 10b illustrates the significant decreases in the CI values between the FCR-FD and ECU-ED muscle unions (*p* = 0.009, EF = 0.119, *F* = 2.00, one-way ANOVA with the Bonferroni *post hoc* test), the ECU-ED muscle union and the BIC muscle (*p* = 0.002, EF = 0.108, *F* = 1.78, one-way ANOVA with the Bonferroni *post hoc* test), the FCR-FD muscle union and the BIC muscle (*p* ≤ 0.001, EF = 0.168, *F* = 2.97, one-way ANOVA with the Bonferroni *post hoc* test), and the BIC and TRI muscle pair (*p* ≤ 0.001, EF = 0.139, *F* = 2.38, one-way ANOVA with the Bonferroni *post hoc* test) during the evaluations across the 20 training sessions. No significant increase or decrease was detected in the EMG parameters of other target muscles and muscle pairs.

## Discussion

The EMG-driven exoneuromusculoskeleton was developed to assist self-help poststroke upper limb training with minimal professional assistance. The pressure/torque transmission properties of the designed musculoskeleton were evaluated, and the assistive capability of the exoneuromusculoskeleton on patients with chronic stroke was assessed using different assistance combinations of NMES and the musculoskeleton. A pilot trial was also conducted to validate the feasibility of device-assisted self-help upper limb rehabilitation.

### Design of exoneuromusculoskeleton

In this study, we integrated soft pneumatic muscles, exoskeleton extension, and NMES in the design of the exoneuromusculoskeleton to assist the upper limb physical practice at the elbow, wrist, and fingers for patients with chronic stroke.

The mechanical support with NMES to the main extensor of a joint was applied in joint extension because upper extremity (UE) extension is more difficult than flexion for most patients after stroke because of the muscle weakness in their affected UE extensors and muscle spasticity in their UE flexors,^46^ which lead to increased resistance in the extension ROM.^74,75^ The results indicated that the elbow musculoskeleton exerted an extension torque of up to 4.3 Nm across the elbow joint when the maximum inner pressure of the musculoskeleton reached 96 kPa (Fig. 6a), which was larger than the reported joint resistance in stroke patients with MAS scores of ≤3 at the elbow.^76^ The hand musculoskeleton could generate a maximal extension torque of 0.093 Nm across the MCP joint of the middle finger when its maximum inner pressure reached 96 kPa (Fig. 6c), which was similar to the reported finger resistance in stroke patients with an MAS score of ≤3 at the fingers.^77^ The musculoskeletons alone could enable the recruited stroke patients with severe-to-moderate upper limb impairments (i.e., 15 < FMA <45) to perform extension at the related joints. It was manifested by evaluating the assistive capability of the system on patients with chronic stroke. The results showed that the ROM values for the elbow and finger joints were significantly higher when using the N0M1 assistance scheme (i.e., the joints assisted by the musculoskeletons only) than when not providing any assistance (N0M0) during joint extension.

Spasticity was defined as motor disorder characterized by a velocity-dependent increase in tonic stretch reflexes with exaggerated tendon jerks, resulting from hyperexcitability of the stretch reflex, as one component of the upper motor neuron syndrome.^46^ It was reported that excessive reflex torque would be exerted when the joint rotation velocity was high, for example, larger than 90°/s in stroke patients with MAS scores of ≤3 at the joint.^51,78^ With the assistance from both the musculoskeleton and the NMES (N1M1), the elbow joint extended with an average angular velocity from 8° to 15°/s (Fig. 7a). In this study, the spasticity at the elbow, wrist, and fingers of the recruited subjects was ≤3 as measured by the MAS, and the angular velocities at the joints were all below 15°/s in the device-assisted motions. Hence, no excessive resistance due to spasticity was generated during the evaluation and training.

In the developed system, the musculoskeletons were attached to the ventral side of the joints and provided torque output to the related joints through inflation. This design is different from most current exoskeletons or soft robotic equipment for UE rehabilitation, in which mechanical assistance is provided from the dorsal side of a joint.^21^ Larger assistive torques are required when providing assistance from the dorsal side of a joint than when providing assistance from its ventral side.^79,80^ Less torque output is also associated with a lower power consumption and more compact size.

The torque-to-weight ratios of the musculoskeleton were 27.2 Nm/kg for the elbow and 9.3 Nm/kg for the fingers. These ratios were comparable with those (7.7–28.3 Nm/kg) reported for other pneumatic soft robots in the literature^29,79,81^ and considerably higher than those of rigid exoskeletons. For example, MyoPro (elbow–wrist–hand exoskeleton), its elbow/hand module, was reported to have a maximal torque-to-weight ratio of 7 Nm/kg for the elbow and 3.4 Nm/kg for the fingers, as well as a total weight of ∼1.8 kg.^82^ In previous studies, pneumatic soft robots were actuated with powerful and heavy compressors, whose weights were not counted in the calculation of the torque-to-weight ratio.^79–81,83^ The miniature compressors used in the developed exoneuromusculoskeleton were the wearable parts of the system. Thus, the total weight on the upper limb was 368 g when the system was fully mounted (Fig. 1). The developed system with a lightweight and wearable design has the potential to support mobile rehabilitation for the upper limb.

In the free loading test, the inner pressure of the elbow musculoskeleton reached close to 100 kPa in <66 s (Fig. 6b) and that of the hand musculoskeleton reached nearly 100 kPa within 17 s (Fig. 6d). The air volume of the pneumatic chamber of the actuator was reduced by the musculoskeleton with mechanical integration of the rigid exoskeleton and pneumatic muscle. Thus, a fast response time was obtained for inflation with the miniature compressors. More powerful and larger compressors were used in soft pneumatic robots in previous studies^79–81,83^ to achieve equivalent mechanical outputs and responses to assist upper limb movements.

In the extension phase, the biological muscle actuation induced by NMES generated additional force for limb extension, which reduced the demand for the external force produced by the musculoskeleton. In this study, wrist extension was only supported by NMES to minimize the size of the mechanical structure of the system. One-channel NMES can support wrist extension with the hand open in clinical practice,^84^ and positive rehabilitation outcomes were observed in a previous study in wrist/hand practice assisted by mechanical support and one-channel NMES of the ECU-ED muscle union.^45^ The assistive capability of the exoneuromusculoskeleton for patients with chronic stroke was also evaluated with different assistance combinations of NMES and the musculoskeleton. The results of the evaluations are discussed in the following section.

### Evaluation of joint assistance by the EMG-driven exoneuromusculoskeleton

Most stroke survivors have a limited ability to perform voluntary joint extension.^85,86^ It is difficult to achieve the ROMs of able-bodied people due to the spasticity at the flexsors,^87^ muscle discoordination of the UE flexors and extensors during extension motion,^74,88^ and weakness at the extensors.^89^ Thus, assisting joint extension to achieve increased ROMs is necessary in poststroke rehabilitation.^61^

In this study, the assistive capability of the EMG-driven exoneuromusculoskeleton for the elbow, wrist, and finger joints was evaluated according to the ROMs achieved for the related joints in the extension phase with different assistance schemes. The assistive performance was evaluated on participants with chronic stroke having severe-to-moderate upper limb impairments. All participants recruited in this work could complete the elbow flexion from 170° to 30° with their residual voluntary effort, together with NMES assistance. Wrist flexion with the hand closed could be achieved through the NMES on the FCR-FD muscle union, together with the residual voluntary effort from the flexors of the wrist and fingers. In the evaluation, all participants could flex their related joints through their own voluntary effort to their initiated position after the extension of the joints.

In the elbow session, the ROM of the elbow joint was significantly higher with mechanical assistance (N0M1 and N1M1) from the system than without assistance (N0M0). This result implied that the elbow ROM was sensitive to the mechanical assistance from the musculoskeleton. Figure 7a indicates that a longer time was required to reach the steady states of the elbow ROMs with mechanical assistance (N0M1 and N1M1) to the elbow than without mechanical assistance (N0M0 and N1M0). It could be related to the interaction between the participants' voluntary motion and the mechanical support from the musculoskeleton (i.e., the pressure/torque transmission rate of the musculoskeleton, Fig. 6b). However, it was also observed that with both NMES and mechanical assistance (N1M1), the elbow ROM values reached their steady state in ∼9 s with an average of joint angle of 166° (at the ninth second), which was considerably shorter than the time required for achieving the full extension of the elbow musculoskeleton with an inner pressure of 96 kPa (i.e., 66 s) under free loading (Fig. 6b). The response time in elbow extension was shortened mainly because of a decreased inner pressure requirement for the elbow musculoskeleton during inflation when the residual voluntary muscle effort was exerted from the participants together with the assistance from NMES to the TRI muscle, when applied the system to the participants. It was also observed that when only mechanical assistance was provided (N0M1), the elbow ROM values reached their steady state in ∼9 s with a relatively smaller joint angle of the elbow compared with that of N1M1 (Fig. 7a). This result implied that the NMES assistance could cause additional extension at the elbow.

In the wrist session, the wrist ROM was significantly larger when providing NMES assistance (N1M0 and N1M1) for the wrist extension than when not providing NMES assistance (N0M1and N0M0). This result implied that NMES assistance considerably influenced the achievement of a significantly larger ROM at the wrist. It is because the wrist movement was only supported by NMES in the designed system. With NMES assistance at the ECU-ED muscle union and mechanical assistance at the fingers (N1M1), the wrist ROM values reached their steady state in ∼6 s with the average angle of wrist extension of 54° (at the 6th second), and the wrist angle finally reached a mean of 56° at 13th second (Fig. 7b). The wrist ROM with both NMES on ECU-ED and the mechanical assistance at the fingers (N1M1) was larger than that with NMES on ECU-ED only (N1M0) (Fig. 7b). The aforementioned results indicated that the mechanical assistance at the fingers could lead to an increased wrist ROM, which was consistent with the finding of a previous study on wrist mobility. The aforementioned study on wrist mobility suggested that the wrist extension angle was lower when the hand was in a closed-fist position than when the fingers were unconstrained.^62^

In the finger session, the ROMs of the MCP and DIP joints of the thumb were significantly larger with mechanical assistance (N0M1 and N1M1) than with no assistance (N0M0) (Fig. 8). This result indicated that the ROM of the thumb was mainly facilitated by the mechanical torque (N0M1 and N1M1). Figure 8 indicates that the ROMs of the MCP, PIP, and DIP joints of the index, middle, ring, and little fingers were significantly larger with mechanical assistance (N1M0, N0M1, and N1M1) than with no assistance (N0M0). The largest finger ROM was achieved when the fingers received both NMES and mechanical assistance (N1M1) (Fig. 8). The maximal finger ROMs were reached within 12 s, which was shorter than the time required for reaching an inner pressure of 96 kPa (i.e., 17 s) under free loading (Fig. 6d). The shortened response time was mainly because of the residual voluntary effort exerted from the finger extensors together with the assistance from NMES to the ECU-ED muscle union.

The results (Table 4) indicated that the participants with severe-to-moderate upper limb impairments could perform limb movements with significantly larger ROMs at the elbow, wrist, and fingers when their affected upper limb was assisted with both NMES and mechanical assistance (N1M1) from the developed system than when using their voluntary effort only (N0M0). This finding was consistent with that in our previous study on the use of a hybrid system of exoskeleton and NMES for poststroke upper limb rehabilitation,^45,90,91^ where the best limb performance was obtained when both mechanical and NMES assistances were provided.

### Self-help upper limb training assisted by the EMG-driven exoneuromusculoskeleton

The feasibility of the proposed self-help rehabilitation training was evaluated, and all the participants completed the training with minimal professional assistance in the laboratory. Close professional assistance was provided only in the first three training sessions. All the participants completed the remaining 17 training sessions independently, and they achieved significant motor improvements in the upper limb after the training. The results obtained in the evaluation sessions indicated that the participants could achieve the largest ROMs with the N1M1 assistance scheme. Therefore, the N1M1 scheme was adopted in the pilot trial. Together with the residual voluntary effort from the paretic limb, the time needed for a cycle of the training task, namely (1) elbow extension, (2) wrist extension with the hand open, (3) wrist flexion with the hand closed, and (4) elbow flexion, was from 40 to 50 s, which was comparable with their natural speed in the paretic upper limb.^45^ With the N1M1, the time needed for performing elbow extension was ∼15 s, wrist extension with the hand open was around 12 s, wrist flexion with the hand closed was ∼6 s, and elbow flexion was around 8 s. It was observed that performing flexions of the related joints was easier and faster than the extensions, since most of the stroke survivors had superior voluntary motion capability in performing joint flexion than extension.^46^

The training improved voluntary motor functions of the entire paretic upper limb and released the muscle spasticity at the elbow, wrist, and fingers. The voluntary motor function recovery of the related joints of the entire paretic upper limb was indicated by the significant increase in the FMA (shoulder/elbow and wrist/hand) scores after the training. These motor function improvements were maintained at the 3-month follow-up. A significant increase was also found in the ARAT scores after the training. This finding not only suggested improved voluntary motor functions of the upper limb but also indicated the recovery of finger function, including grasping, gripping, and pinching movements with fine precision control of the fingers.

Stroke survivors usually exhibit muscle discoordination due to muscle spasticity and compensatory motions in the affected limb.^9^ The release of flexor spasticity in the elbow, wrist, and fingers was found after the training, as revealed by the significant decrease in the MAS scores at the related joints after the training. The decrease in the MAS scores after the training also suggested improved muscle coordination and control of synergic muscle activity in proximal and distal joints.^92^ With NMES assistance for the extensors, the muscle spasticity at the elbow, wrist, and fingers was effectively reduced. This finding was consistent with those of clinical trials on NMES-assisted poststroke rehabilitation.^39,40,93^

Limb practice with close-to-normal muscular coordination and minimized compensatory motions was achieved through the combined assistance of NMES and mechanical torque in the joint extension phases.^39,45^ Such limb practice led to a reduction in excessive muscle activities and superior muscle coordination, as revealed by the decrease in the EMG activation levels at the flexors, cocontractions between the antagonist muscle pairs related to the wrist/hand and elbow, and cocontractions between the elbow flexor and the distal joints. The significant decrease in the EMG activation levels of the FCR-FD muscle union and BIC muscle indicated a reduction in excessive muscle activities of the related muscles during the arm reaching and withdrawing as well as the hand opening and grasping motions, which suggested that the muscle spasticity of the related joints was reduced (manifested in the significantly decreased MAS scores at the elbow, wrist, and fingers after training). The CIs revealed the recovery of muscular coordination and the muscular coactivity within a joint or across joints in the upper limb.^71,72^ The significant decreases in the CIs of the FCR-FD and ECU-ED muscle unions and the BIC and TRI muscle pair indicated that the muscle coordination for achieving the reaching and withdrawing motions through the flexion and extension at the elbow, wrist, and finger joints was improved after training. Various compensatory movements from the proximal joints were observed during motions at the distal joints for patients with stroke.^4,84^ These compensatory movements can result in excessive cocontractions in the muscles related to the proximal and distal joints. The significantly decreased CIs between the ECU-ED muscle union and BIC muscle and between the FCR-FD muscle union and BIC muscle indicated a reduction in the coactivities between cross-joint muscles during limb motions, improvements in isolation of the wrist and finger movements from the elbow movements. It implied that compensation movements from cocontraction on the proximal joint during distal joint motions were reduced.

Conclusive EMG results were found in (1) the proximal and distal flexors, i.e., significant decreased in the EMG activation level for the BIC and FCR-FD, (2) the proximal and distal antagonist muscle pairs, that is, significantly decreased in the CI values between the BIC and TRI, and the FCR-FD and ECU-ED, and (3) cross-joint muscles, that is, significantly decreased in the CI values between the ECU-ED and BIC, and the FCR-FD and BIC. These results indicated a reduction in excessive muscle activities in the flexors, mainly related to the release of spasticity, and a reduction in cocontraction between a muscle pair. The nonsignificant EMG parameters were mainly related to the ECU-ED and TRI, which could be related to the weakness in these extensors,^46,89^ or a small sample size in this work. In our future work, large-scale, randomized-controlled trials will be conducted.

It was understood that the treatment with NMES could induce fatigue in a muscle due to the reversed recruiting sequence of muscle fibers in comparison with that during voluntary muscle contractions.^31^ Mean frequency drop in EMG was used for monitoring the process of muscle fatigue.^94^ We compared the mean frequencies of the EMG signals of the driving muscles in a session, that is, EMG signals used for the triggering control. The average mean frequency variation in a session was ≤5%, which could be considered as the muscles were not fatigued in the training.^95^ Furthermore, a 15-min break was provided between two consecutive 30-min practice to prevent muscle fatigue. NMES-induced possible muscle fatigue was minimized during the training of this study.

After the EMG-driven exoneuromusculoskeleton assisted self-help upper limb training, all the participants exhibited improved motor functions, reduced muscle spasticity, and superior muscle coordination associated with significantly improved clinical scores and cross-session-recorded EMG parameters. These results suggested that coordinated multijoint limb practice with the designed assistive function of NMES and the musculoskeleton can facilitate effective motor recovery of stroke patients with severe-to-moderate upper limb impairments.

## Conclusions

In this study, a novel EMG-driven exoneuromusculoskeleton was designed for supporting self-help poststroke upper limb rehabilitation with minimum professional assistance. The developed system could assist intensive and repeated upper limb practice at the elbow, wrist, and fingers under the voluntary intention control by residual voluntary EMG signals from the affected upper limb, with a lightweight, compact, and low-power requirement design. The results indicated that the largest ROMs were achieved when the related joints were provided both NMES and mechanical assistance. The participants (patients with chronic stroke) could complete the self-help device-assisted training with minimal professional assistance (i.e., assistance on the training setup and device operation was provided only in the first three training sessions). When adopting the optimal NMES and robot assistance scheme, the EMG-driven exoneuromusculoskeleton-assisted rehabilitation program could facilitate motor improvement in the affected upper limb of the participants with chronic stroke. After a 20-session device-assisted training, significant motor improvements were achieved, including improved voluntary motor functions in the entire upper limb; released muscle spasticity at the elbow, wrist, and fingers; and improved muscular coordination in the entire upper limb.

## References

[1] Kwakkel G, Kollen BJ, van der Grond J, *et al.* Probability of regaining dexterity in the flaccid upper limb: impact of severity of paresis and time since onset in acute stroke. Stroke 2003;34:2181–2186. 12907818 10.1161/01.STR.0000087172.16305.CD

[2] Kong KH, Chua KS, Lee J. Recovery of upper limb dexterity in patients more than 1 year after stroke: frequency, clinical correlates and predictors. Neurorehabilitation 2011;28:105–111. 21447911 10.3233/NRE-2011-0639

[3] Langhorne P, Bernhardt J, Kwakkel G. Stroke rehabilitation. Lancet 2011;377:1693–1702. 21571152 10.1016/S0140-6736(11)60325-5

[4] Good DC, Bettermann K, Reichwein RK. Stroke rehabilitation. Continuum Lifelong Learn Neurol 2011;17:545–567. 10.1212/01.CON.0000399072.61943.38 22810867

[5] Harris JE, Eng JJ. Strength training improves upper-limb function in individuals with stroke: a meta-analysis. Stroke 2010;41:136–140. 19940277 10.1161/STROKEAHA.109.567438

[6] Sun J, Ke Z, Yip SP, *et al.* Gradually increased training intensity benefits rehabilitation outcome after stroke by BDNF upregulation and stress suppression. Biomed Res Int 2014;2014:925762. 25045713 10.1155/2014/925762 PMC4090448

[7] Farmer J, Zhao X, Van Praag H, *et al.* Effects of voluntary exercise on synaptic plasticity and gene expression in the dentate gyrus of adult male Sprague–Dawley rats in vivo. Neuroscience 2004;124:71–79. 14960340 10.1016/j.neuroscience.2003.09.029

[8] Volpe BT, Ferraro M, Lynch D, *et al.* Robotics and other devices in the treatment of patients recovering from stroke. Curr Neurol Neurosci Rep 2005;5:465–470. 16263058 10.1007/s11910-005-0035-y

[9] Dewald JPA, Sheshadri V, Dawson ML, *et al.* Upper-limb discoordination in hemiparetic stroke: implications for neurorehabilitation. Top Stroke Rehabil 2001;8:1–12. 10.1310/WA7K-NGDF-NHKK-JAGD 14523747

[10] Woo J, Chan SY, Sum MWC, *et al.* In patient stroke rehabilitation efficiency: influence of organization of service delivery and staff numbers. BMC Health Serv Res 2008;8:86. 18416858 10.1186/1472-6963-8-86 PMC2391159

[11] Maciejasz P, Eschweiler J, Gerlach-Hahn K, *et al.* A survey on robotic devices for upper limb rehabilitation. J Neuroeng Rehabil 2014;11:3. 24401110 10.1186/1743-0003-11-3 PMC4029785

[12] Steinhubl SR, Muse ED, Topol EJ. The emerging field of mobile health. Sci Transl Med 2015;7:283rv3. 10.1126/scitranslmed.aaa3487 PMC4748838 25877894

[13] Chang WH, Kim YH. Robot-assisted therapy in stroke rehabilitation. J Stroke 2013;15:174. 24396811 10.5853/jos.2013.15.3.174 PMC3859002

[14] Islam M, Spiewak C, Rahman M, *et al.* A brief review on robotic exoskeletons for upper extremity rehabilitation to find the gap between research porotype and commercial type. Adv Robot Autom 2017;6:2.

[15] Manna SK, Dubey VN. Comparative study of actuation systems for portable upper limb exoskeletons. Med Eng Phys 2018;60:1–13. 30122472 10.1016/j.medengphy.2018.07.017

[16] Molteni F, Gasperini G, Cannaviello G, *et al.* Exoskeleton and end-effector robots for upper and lower limbs rehabilitation: narrative review. PM&R 2018;10:S174–S188. 30269804 10.1016/j.pmrj.2018.06.005

[17] Yap HK, Lim JH, Nasrallah F, *et al.* Characterisation and evaluation of soft elastomeric actuators for hand assistive and rehabilitation applications. J Med Eng Technol 2016;40:199–209. 27007297 10.3109/03091902.2016.1161853

[18] Roh J, Rymer WZ, Perreault EJ, *et al.* Alterations in upper limb muscle synergy structure in chronic stroke survivors. J Neurophysiol 2013;109:768–781. 23155178 10.1152/jn.00670.2012 PMC3567389

[19] Xiloyannis M, Chiaradia D, Frisoli A, *et al.* Physiological and kinematic effects of a soft exosuit on arm movements. J Neuroeng Rehabil 2019;16:29. 30791919 10.1186/s12984-019-0495-y PMC6385456

[20] Shahid T, Gouwanda D, Nurzaman SG. Moving toward soft robotics: a decade review of the design of hand exoskeletons. Biomimetics 2018;3:17. 10.3390/biomimetics3030017 PMC6352684 31105239

[21] Chu CY, Patterson RM. Soft robotic devices for hand rehabilitation and assistance: a narrative review. J Neuroeng Rehabil 2018;15:9. 29454392 10.1186/s12984-018-0350-6 PMC5816520

[22] Bartlett NW, Tolley MT, Overvelde JT, *et al.* A 3D-printed, functionally graded soft robot powered by combustion. Science 2015;349:161–165. 26160940 10.1126/science.aab0129

[23] Gull MA, Bai S, Bak T. A review on design of upper limb exoskeletons. Robotics 2020;9:16.

[24] Zatopa A, Walker S, Menguc Y. Fully soft 3D-printed electroactive fluidic valve for soft hydraulic robots. Soft Robot 2018;5:258–271. 29608429 10.1089/soro.2017.0019

[25] Lessard S, Pansodtee P, Robbins A, *et al.* A soft exosuit for flexible upper-extremity rehabilitation. IEEE Trans Neural Syst Rehabil Eng 2018;26:1604–1617. 29994617 10.1109/TNSRE.2018.2854219

[26] Cho HS, Kim TH, Hong TH, *et al.* Ratchet-integrated pneumatic actuator (RIPA): a large-stroke soft linear actuator inspired by sarcomere muscle contraction. Bioinspir Biomim 2020;15:036011. 32069446 10.1088/1748-3190/ab7762

[27] Wehner M, Tolley MT, Mengüç Y, *et al.* Pneumatic energy sources for autonomous and wearable soft robotics. Soft Robot 2014;1:263–274.

[28] Park Y-L, Chen B-r, Pérez-Arancibia NO, *et al.* Design and control of a bio-inspired soft wearable robotic device for ankle–foot rehabilitation. Bioinspir Biomim 2014;9:016007. 24434598 10.1088/1748-3182/9/1/016007

[29] Bartlett NW, Lyau V, Raiford WA, *et al.* A soft robotic orthosis for wrist rehabilitation. J Med Device 2015;9:1–3.

[30] Balasubramanian S, He J. Adaptive control of a wearable exoskeleton for upper-extremity neurorehabilitation. Appl Bionics Biomech 2012;9:99–115.

[31] Chae J, Sheffler L, Knutson J. Neuromuscular electrical stimulation for motor restoration in hemiplegia. Top Stroke Rehabil 2008;15:412–426. 19008202 10.1310/tsr1505-412

[32] Freeman CT, Hughes A-M, Burridge JH, *et al.* Iterative learning control of FES applied to the upper extremity for rehabilitation. Control Eng Pract 2009;17:368–381.

[33] Takeda K, Tanino G, Miyasaka H. Review of devices used in neuromuscular electrical stimulation for stroke rehabilitation. Med Devices (Auckl) 2017;10:207. 28883745 10.2147/MDER.S123464 PMC5576704

[34] Cheung VC, Niu CM, Li S, *et al.* A novel FES strategy for poststroke rehabilitation based on the natural organization of neuromuscular control. IEEE Rev Biomed Eng 2018;12:154–167. 30307876 10.1109/RBME.2018.2874132

[35] Resquín F, Gómez AC, Gonzalez-Vargas J, *et al.* Hybrid robotic systems for upper limb rehabilitation after stroke: a review. Med Eng Phys 2016;38:1279–1288. 27692878 10.1016/j.medengphy.2016.09.001

[36] Rodgers MM, Alon G, Pai VM, *et al.* Wearable technologies for active living and rehabilitation: current research challenges and future opportunities. J Rehabil Assist Technol Eng 2019;6:2055668319839607. 31245033 10.1177/2055668319839607 PMC6582279

[37] Basteris A, Nijenhuis SM, Stienen AH, *et al.* Training modalities in robot-mediated upper limb rehabilitation in stroke: a framework for classification based on a systematic review. J Neuroeng Rehabil 2014;11:111. 25012864 10.1186/1743-0003-11-111 PMC4108977

[38] Hu XL, Tong KY, Song R, *et al.* A comparison between electromyography-driven robot and passive motion device on wrist rehabilitation for chronic stroke. Neurorehabil Neural Repair 2009;23:837–846. 19531605 10.1177/1545968309338191

[39] Nam C, Rong W, Li W, *et al.* The effects of upper-limb training assisted with an electromyography-driven neuromuscular electrical stimulation robotic hand on chronic stroke. Front Neurol 2017;8:679. 29312116 10.3389/fneur.2017.00679 PMC5735084

[40] Qiuyang Q, Nam C, Guo Z, *et al.* Distal versus proximal-an investigation on different supportive strategies by robots for upper limb rehabilitation after stroke: a randomized controlled trial. J Neuroeng Rehabil 2019;16:64. 31159822 10.1186/s12984-019-0537-5 PMC6545723

[41] Muraoka Y. Development of an EMG recording device from stimulation electrodes for functional electrical stimulation. Front Med Biol Eng 2002;11:323–333. 12735431 10.1163/156855701321138969

[42] Breen PP, Corley GJ, O'Keeffe DT, *et al.* A programmable and portable NMES device for drop foot correction and blood flow assist applications. Med Eng Phys 2009;31:400–408. 18667351 10.1016/j.medengphy.2008.05.003

[43] Ho SK, Tong RKY, Chen M, *et al.* Hand rehabilitation robot using electromyography. In: Tong RKY, eds. Biomechatronics in Medicine and Health Care. Pan Stanford Publishing Pte. Ltd., 2011, pp. 77–92.

[44] Hu XL, Tong KY, Wei XJ, *et al.* The effects of post-stroke upper-limb training with an electromyography (EMG)-driven hand robot. J Electromyogr Kinesiol 2013;23:1065–1074. 23932795 10.1016/j.jelekin.2013.07.007

[45] Rong W, Li WM, Pang MK, *et al.* A Neuromuscular Electrical Stimulation (NMES) and robot hybrid system for multi-joint coordinated upper limb rehabilitation after stroke. J Neuroeng Rehabil 2017;14:34. 28446181 10.1186/s12984-017-0245-y PMC5406922

[46] Bhakta BB. Management of spasticity in stroke. Br Med Bull 2000;56:476–485. 11092096 10.1258/0007142001903111

[47] Hu XL, Tong KY, Hu JY, *et al.* Wearable robotic device with bracing system with moisture and pressure management for comfortable rehabilitation, U.S.A., 14/093066, CN104666048 China patent granted. 2017.

[48] Liu R, Little T, Williams J. Compression form-fitted athletic wear: pressure performance, moisture management properties under different tension ratios, and corresponding psychophysical responses. Fiber Polym 2014;15:632–644.

[49] Dawal SZM, Ismail Z, Yusuf K, *et al.* Determination of the significant anthropometry dimensions for user-friendly designs of domestic furniture and appliances–Experience from a study in Malaysia. Measurement 2015;59:205–215.

[50] Jee SC, Yun MH. An anthropometric survey of Korean hand and hand shape types. Int J Ind Ergon 2016;53:10–18.

[51] Bhadane MY, Gao F, Francisco GE, *et al.* Correlation of resting elbow angle with spasticity in chronic stroke survivors. Front Neurol 2015;6:183. 26379617 10.3389/fneur.2015.00183 PMC4549629

[52] Koh TH, Cheng N, Yap HK, *et al.* Design of a soft robotic elbow sleeve with passive and intent-controlled actuation. Front Neurosci 2017;11:597. 29118693 10.3389/fnins.2017.00597 PMC5660967

[53] Duncan SF, Saracevic CE, Kakinoki R. Biomechanics of the hand. Hand Clin 2013;29:483–492. 24209947 10.1016/j.hcl.2013.08.003

[54] Ashwoth B. Preliminary trial of carisoprodol in multiple sclerosis. Practitioner 1964;192:540–542. 14143329

[55] Fugl-Meyer AR, Jääskö L, Leyman I, *et al.* The post-stroke hemiplegic patient. 1. A method for evaluation of physical performance. Scand J Rehabil Med 1975;7:13–31. 1135616

[56] Folstein MF, Folstein SE, McHugh PR. “Mini-mental state.” A practical method for grading the cognitive state of patients for the clinician. J Psychiatr Res 1975;12:189–198. 1202204 10.1016/0022-3956(75)90026-6

[57] Cutti AG, Paolini G, Troncossi M, *et al.* Soft tissue artefact assessment in humeral axial rotation. Gait Posture 2005;21:341–349. 15760751 10.1016/j.gaitpost.2004.04.001

[58] Hingtgen B, McGuire JR, Wang M, *et al.* An upper extremity kinematic model for evaluation of hemiparetic stroke. J Biomech 2006;39:681–688. 16439237 10.1016/j.jbiomech.2005.01.008

[59] Oh HS, Kim EJ, Kim DY, *et al.* Effects of adjuvant mental practice on affected upper limb function following a stroke: results of three-dimensional motion analysis, fugl-meyer assessment of the upper extremity and motor activity logs. Ann Rehabil Med 2016;40:401. 27446776 10.5535/arm.2016.40.3.401 PMC4951358

[60] Murray IA. Determining upper limb kinematics and dynamics during everyday tasks. Newcastle upon Tyne, England: [Doctoral dissertation]. Newcastle University, 1999.

[61] Lang CE, DeJong SL, Beebe JA. Recovery of thumb and finger extension and its relation to grasp performance after stroke. J Neurophysiol 2009;102:451–459. 19458140 10.1152/jn.91310.2008 PMC2712280

[62] Gehrmann SV, Kaufmann RA, Li ZM. Wrist circumduction reduced by finger constraints. J Hand Surg 2008;33:1287–1292. 10.1016/j.jhsa.2008.04.034 18929190

[63] Clarkson HM. Musculoskeletal Assessment: Joint Range of Motion and Manual Muscle Strength. Baltimore, MD: Lippincott Williams & Wilkins, 2000.

[64] Krebs HI, Volpe BT. Rehabilitation robotics. In: Barnes MP, Good DC, eds. Handbook of Clinical Neurology. Amsterdam, Netherlands: Elsevier, 2013, pp. 283–294. 10.1016/B978-0-444-52901-5.00023-X PMC4688009 23312648

[65] Carroll D. A quantitative test of upper extremity function. J Chronic Dis 1965;18:479–491. 14293031 10.1016/0021-9681(65)90030-5

[66] Wolf SL, Lecraw DE, Barton LA, *et al.* Forced use of hemiplegic upper extremities to reverse the effect of learned nonuse among chronic stroke and head-injured patients. Exp Neurol 1989;104:125–132. 2707361 10.1016/s0014-4886(89)80005-6

[67] Wallace D, Duncan PW, Lai SM. Comparison of the responsiveness of the Barthel Index and the motor component of the Functional Independence Measure in stroke: the impact of using different methods for measuring responsiveness. J Clin Epidemiol 2002;55:922–928. 12393081 10.1016/s0895-4356(02)00410-9

[68] Dewald JP, Pope PS, Given JD, *et al.* Abnormal muscle coactivation patterns during isometric torque generation at the elbow and shoulder in hemiparetic subjects. Brain 1995;118:495–510. 7735890 10.1093/brain/118.2.495

[69] Qian Q, Hu X, Lai Q, *et al.* Early stroke rehabilitation of the upper limb assisted with an electromyography-driven neuromuscular electrical stimulation-robotic arm. Front Neurol 2017;8:447. 28928706 10.3389/fneur.2017.00447 PMC5591334

[70] Hu XL, Tong RK, Ho NSK, *et al.* Wrist rehabilitation assisted by an electromyography-driven neuromuscular electrical stimulation robot after stroke. Neurorehabil Neural Repair 2015;29:767–776. 25549656 10.1177/1545968314565510

[71] Hu XL, Tong KY, Song R, *et al.* Variation of muscle coactivation patterns in chronic stroke during robot-assisted elbow training. Arch Phys Med Rehabil 2007;88:1022–1029. 17678665 10.1016/j.apmr.2007.05.006

[72] Hu XL, Tong KY, Song R, *et al.* Quantitative evaluation of motor functional recovery process in chronic stroke patients during robot-assisted wrist training. J Electromyogr Kinesiol 2009;19:639–650. 18490177 10.1016/j.jelekin.2008.04.002

[73] Razali NM, Wah YB. Power comparisons of shapiro-wilk, kolmogorov-smirnov, lilliefors and anderson-darling tests. J Stat Model Anal 2011;2:21–33.

[74] Halar EM, Stolov WC, Venkatesh B, *et al.* Gastrocnemius muscle belly and tendon length in stroke patients and able-bodied persons. Arch Phys Med Rehabil 1978;59:476–484. 718411

[75] Wood KS, Daluiski A. Management of joint contractures in the spastic upper extremity. Hand Clin 2018;34:517–528. 30286966 10.1016/j.hcl.2018.06.011

[76] Ardabili NS, Abdollahi I, Khorramymehr S, *et al.* Quantitative evaluation of spasticity at the elbow of stroke patients. In: Proceedings of the 2011 18th Iranian Conference of Biomedical Engineering (ICBME). IEEE, 2011:131–136, Tehran, Iran.

[77] Kamper DG, Rymer WZ. Quantitative features of the stretch response of extrinsic finger muscles in hemiparetic stroke. Muscle Nerve 2000;23:954–961. 10842274 10.1002/(sici)1097-4598(200006)23:6<954::aid-mus17>3.0.co;2-0

[78] Condliffe EG, Clark DJ, Patten C. Reliability of elbow stretch reflex assessment in chronic post-stroke hemiparesis. Clin Neurophysiol 2005;116:1870–1878. 15979400 10.1016/j.clinph.2005.02.030

[79] Yap HK, Lim JH, Goh JCH, *et al.* Design of a soft robotic glove for hand rehabilitation of stroke patients with clenched fist deformity using inflatable plastic actuators. J Med Device 2016;10:044504.

[80] Connelly L, Jia Y, Toro ML, *et al.* A pneumatic glove and immersive virtual reality environment for hand rehabilitative training after stroke. IEEE Trans Neural Syst Rehabil Eng 2010;18:551–559. 20378482 10.1109/TNSRE.2010.2047588

[81] Oguntosin V, Harwin WS, Kawamura S, *et al.* Development of a wearable assistive soft robotic device for elbow rehabilitation. In: Proceedings of the 2015 IEEE International Conference on Rehabilitation Robotics (ICORR). IEEE, 2015:747–752, Singapore.

[82] Dunaway S, Dezsi DB, Perkins J, *et al.* Case report on the use of a custom myoelectric elbow–wrist–hand orthosis for the remediation of upper extremity paresis and loss of function in chronic stroke. Mil Med 2017;182:e1963–e1968. 28810999 10.7205/MILMED-D-16-00399

[83] Wilkening A, Stöppler H, Ivlev O. Adaptive assistive control of a soft elbow trainer with self-alignment using pneumatic bending joint. In: Proceedings of the 2015 IEEE International Conference on Rehabilitation Robotics (ICORR). IEEE, 2015:729–734, Singapore.

[84] Raghavan P. Upper limb motor impairment after stroke. Phys Med Rehabil Clin N Am 2015;26:599–610. 26522900 10.1016/j.pmr.2015.06.008 PMC4844548

[85] O'dwyer NJ, Ada L, Neilson PD. Spasticity and muscle contracture following stroke. Brain 1996;119:1737–1749. 8931594 10.1093/brain/119.5.1737

[86] Williams PE. Effect of intermittent stretch on immobilised muscle. Ann Rheum Dis 1988;47:1014–1016. 3207382 10.1136/ard.47.12.1014 PMC1003657

[87] Cozens JA. Robotic assistance of an active upper limb exercise in neurologically impaired patients. IEEE Trans Rehabil Eng 1999;7:254–256. 10391596 10.1109/86.769416

[88] Kamper DG, Rymer WZ. Impairment of voluntary control of finger motion following stroke: role of inappropriate muscle coactivation. Muscle Nerve 2001;24:673–681. 11317278 10.1002/mus.1054

[89] Canning CG, Ada L, O'Dwyer NJ. Abnormal muscle activation characteristics associated with loss of dexterity after stroke. J Neurol Sci 2000;176:45–56. 10865092 10.1016/s0022-510x(00)00305-1

[90] Hu XL, Tong KY, Li R, *et al.* The effects of electromechanical wrist robot assistive system with neuromuscular electrical stimulation for stroke rehabilitation. J Electromyogr Kinesiol 2012;22:431–439. 22277205 10.1016/j.jelekin.2011.12.010

[91] Rong W, Tong KY, Hu XL, *et al.* Effects of electromyography-driven robot-aided hand training with neuromuscular electrical stimulation on hand control performance after chronic stroke. Disabil Rehabil Assist Technol 2015;10:149–159. 24377757 10.3109/17483107.2013.873491

[92] Chae J, Yang G, Park BK, *et al.* Muscle weakness and cocontraction in upper limb hemiparesis: relationship to motor impairment and physical disability. Neurorehabil Neural Repair 2002;16:241–248. 12234087 10.1177/154596830201600303

[93] Boyaci A, Topuz O, Alkan H, *et al.* Comparison of the effectiveness of active and passive neuromuscular electrical stimulation of hemiplegic upper extremities: a randomized, controlled trial. Int J Rehabil Res 2013;36:315–322. 23579106 10.1097/MRR.0b013e328360e541

[94] Edman K. Myofibrillar fatigue versus failure of activation. In: Gandevia SC, Enoka RM, McComas AJ, *et al.*, eds. Proceedings of the Fatigue. Boston, MA, USA: Springer, 1995:29–43. 10.1007/978-1-4899-1016-5_3 8585458

[95] Allison G, Fujiwara T. The relationship between EMG median frequency and low frequency band amplitude changes at different levels of muscle capacity. Clin Biomech 2002;17:464–469. 10.1016/s0268-0033(02)00033-5 12135548

